# Smarcc1 drives optic stalk patterning and optic nerve head astrocyte differentiation

**DOI:** 10.1242/dev.205769

**Published:** 2026-08-13

**Authors:** Nitay Zuk-Bar, Shai Ovadia, Guizhong Cui, Alexey Obolensky, Eyal Banin, Ron Ofri, Naihe Jing, Ruth Ashery-Padan

**Affiliations:** ^1^ Department of Human Molecular Genetics and Biochemistry, Gray Faculty of Medical & Health Sciences and Sagol School of Neurosciences, Tel Aviv University, Tel Aviv 69978, Israel; ^2^ Guangzhou National Laboratory, School of Basic Medical Sciences, Guangzhou Medical University, Guangzhou 510005, China; ^3^ Faculty of Medicine, Center for Retinal and Macular Degenerations, Department of Ophthalmology, Hadassah Medical Center and Hebrew University Faculty of Medicine, Jerusalem 91120, Israel; ^4^ Faculty of Agriculture, Food and Environment, Hebrew University of Jerusalem, Rehovot 7610001, Israel

**Keywords:** Optic stalk, Optic nerve head, SWI/SNF, Eye development, Mouse

## Abstract

The optic nerve develops from the neuroectodermal optic stalk, which undergoes coordinated morphogenesis and gives rise to optic nerve astrocytes that support retinal ganglion cell axons. Here, we define the progression of astrocyte formation from the optic stalk and identify stage-specific functions of the SWI/SNF scaffolding subunits Smarcc1 and Smarcc2. Both factors are co-expressed in retinal pigment epithelium (RPE) and optic stalk progenitors, with Smarcc2 persisting in differentiated RPE and astrocytes. Conditional deletion using *Dct-Cre* revealed compensatory activity in pigmented lineages, whereas Smarcc1 loss uniquely disrupted optic nerve head morphogenesis, resulting in glial lamina collapse, retinal ganglion cell degeneration and progressive visual decline. Spatial transcriptomics and functional assays show that Smarcc1 enables dorsal optic stalk progenitors to transition from a pigmented, RPE-like state to astrocyte progenitors by repressing pigment gene programs and permitting Pax2 and Sox2 activity. After specification, Smarcc1 is also required for glial lamina assembly and astrocyte migration into the inner retina. These findings demonstrate that Smarcc1-dependent chromatin remodeling coordinates astrocyte specification with optic nerve head morphogenesis to maintain long-term retinal function.

## Teaser

**Summary:** Conditional deletion of Smarcc1 and spatial transcriptomics reveal a stage-specific role for Smarcc1 in the specification and differentiation of optic nerve astrocytes and identify interactions with transcription factor networks driving optic stalk patterning in mouse.

## INTRODUCTION

Complex interactions between transcription factors (TFs) and chromatin remodeling complexes govern the stage- and cell-specific gene expression programs required for organ formation. In the central nervous system, this interplay is exemplified by the transition of neuroectodermal progenitors into diverse neural and glial lineages. A striking case is the optic nerve, which arises from the transient neuroepithelial optic stalk (OS). Initially serving as a conduit for retinal ganglion cell (RGC) axons extending to the brain, the OS later differentiates into astrocytes that support and ensheathe these axons. Although the broad progression from OS progenitors to type I astrocytes has been described, the mechanisms that establish OS regional identity and direct progenitors toward an astroglial fate remain incompletely understood.

The optic nerve transmits visual information through RGC axons and is supported by astrocytes and a vascular network (Butt et al., 2004). At its anterior end, the optic nerve head (ONH) serves as the convergence point of all RGC axons and maintains them in an unmyelinated state. This region includes the optic disc within the eye cup and the optic nerve laminar region posterior to it (Morcillo et al., 2006; Sun et al., 2009; Bernstein et al., 2020). The ONH also contains Sox2-positive neural precursors that contribute to postnatal optic nerve extension (Bernstein et al., 2020). The ONH integrity is crucial for RGC survival throughout life, and structural changes in this region are linked to glaucomatous optic neuropathy (Howell et al., 2007; Pitha et al., 2024). These features underscore the need to understand how OS cells acquire the astroglial identity necessary for proper optic nerve formation and lifelong axonal support.

During embryogenesis, the OS originates from the proximal optic vesicle, a neuroectodermal outgrowth of the forebrain (Colello and Guillery, 1992). The distal vesicle invaginates to form the optic cup, which consists of an outer layer of the progenitors for the presumptive retinal pigmented epithelium (pRPE) and an inner layer of retinal progenitor cells. The proximal region of the optic vesicle narrows into the epithelial tube of the OS. A ventral fissure allows entry of hyaloid vasculature and mesenchymal cells (Barishak, 2001; Saint-Geniez and D'Amore, 2004; Lutty and McLeod, 2018), which ultimately close as the fissure fuses to form a continuous tube ensheathing emerging RGC axons (Silver and Robb, 1979). Over developmental time, OS progenitors delaminate and differentiate into type I astrocytes (Heavner and Pevny, 2012; Tao and Zhang, 2014; Cardozo et al., 2020). Yet how regional identity is established within the OS, and how progenitors are directed toward an astroglial fate, remain mostly unresolved.

Patterning of the OS and optic cup arises from positional cues – Shh at the midline, Tgfβ from mesenchyme and Fgfs/WNTs from surface ectoderm (Martínez-Morales et al., 2004; Morcillo et al., 2006; Cardozo et al., 2020). These factors instruct the expression of a combination of region-specific TFs, which in turn trigger the gene regulatory networks required for regional cell fates and, at the same time, inhibit competing developmental programs (Martínez-Morales et al., 2004; Patel and Sowden, 2019; Cardozo et al., 2020). A well-characterized example is the mutual antagonism between Pax2 and Pax6, which establishes the stalk–cup boundary and restricts OS cells to astroglial fates. Loss of Pax2 disrupts the stalk–cup boundary, causes Pax6 upregulation in stalk cells and prevents optic fissure closure, resulting in coloboma (Schwarz et al., 2000; Bosze et al., 2021). Although TF interplay has been well studied in this context, much less is known about how chromatin remodeling complexes contribute to stalk patterning and astrocyte specification.

The SWI/SNF (BAF) complexes are essential regulators of neural development, promoting chromatin accessibility in opposition to Polycomb-mediated repression (Ho and Crabtree, 2010; Kadoch et al., 2017). Their core subunits – Smarca4/2 (Brg1/Brm), Smarcb1 (Baf47) and Smarcc1/2 (Baf155/170) – interact with lineage-restricted TFs to direct fate specification (Tuoc et al., 2013b; Hoffman et al., 2018). Although Smarcc1 and Smarcc2 act as interchangeable scaffold components, they also exhibit non-redundant developmental functions: Smarcc1 is essential for embryonic stem cell pluripotency (Ho et al., 2009), and both subunits modulate cortical development by altering Pax6 transcriptional activity (Tuoc et al., 2013a). SWI/SNF activity has also been linked to Sox2 and Mitf function in diverse settings (Ahmed et al., 2012; Laurette et al., 2015), and conditional removal of both Smarcc1 and Smarcc2 in the RPE redirects cells to neural-like states (Ovadia et al., 2023). Smarcc1 and Smarcc2 are implicated in a group of neurodevelopmental disorders collectively termed BAFopathies (Chen et al., 2022), underscoring the importance of dissecting their individual roles, compensatory mechanisms, and functional specificity. Despite these insights, how Smarcc1 and Smarcc2 coordinate with TFs to establish OS identity and promote astrocyte differentiation remains unresolved.

Here, we investigate the contributions of Smarcc1 and Smarcc2 to early optic nerve development. We identify a temporally restricted requirement for Smarcc1 in the specification and differentiation of optic nerve astrocytes and delineate how SWI/SNF activity integrates with TF networks to shape OS regional identity. These findings address a key gap in our understanding of optic nerve development and reveal new principles of chromatin–TF interplay in the central nervous system.

## RESULTS

### Distinct stage- and cell-specific expression of Smarcc1 and Smarcc2 in the optic vesicle lineages

To investigate the stage- and tissue-specific roles of Smarcc1 and Smarcc2 in the pigmented lineages of the optic cup, we characterized, by antibody labeling, their expression patterns in the neuroectodermal derivatives of the embryonic optic cup.

**Fig. 1.**
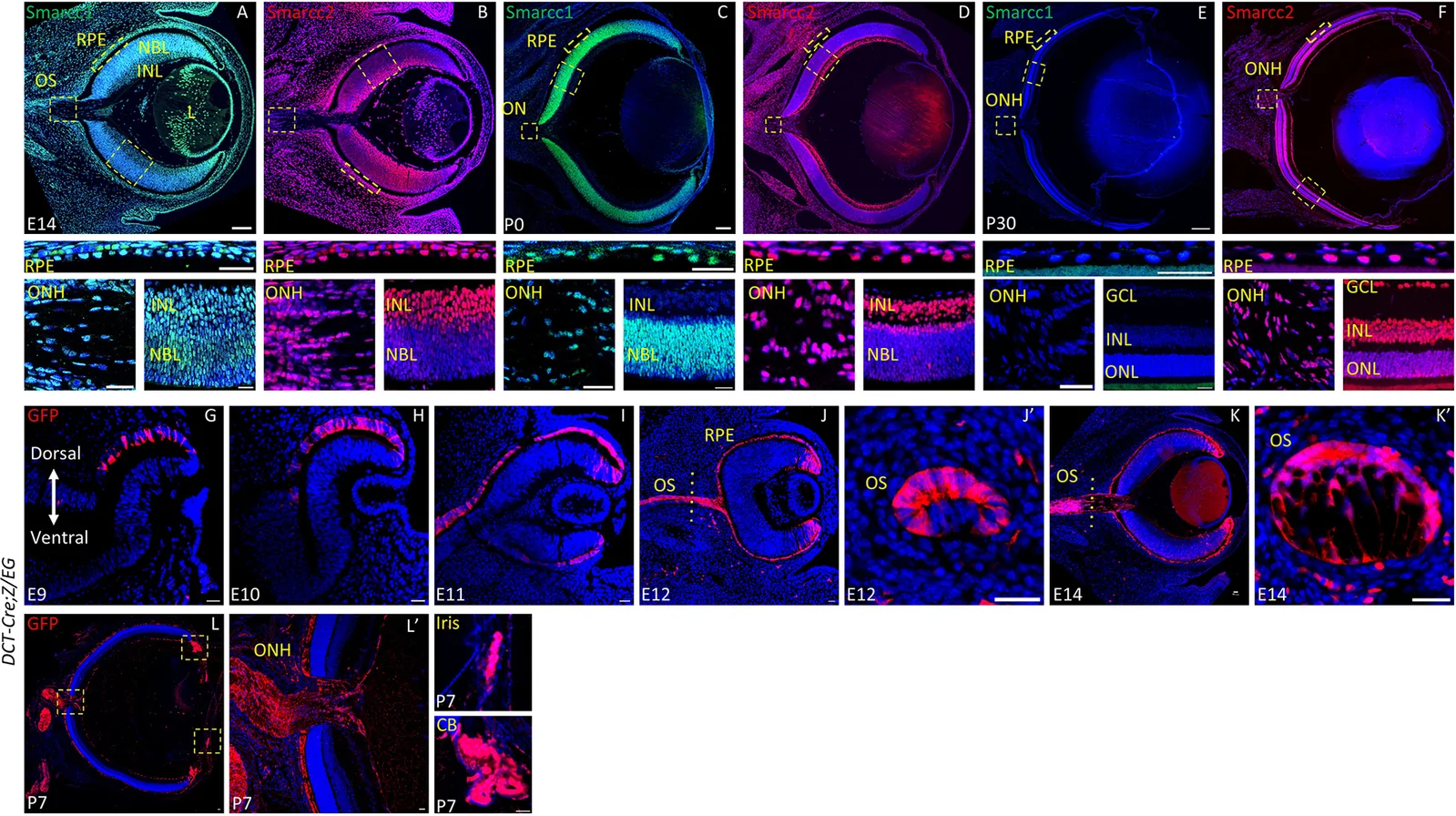
**Stage-specific expression of Smarcc1 and Smarcc2 in the optic vesicle-derived lineages and characterization of *Dct-Cre* activity.** (A-F) Immunolabeling of E14 (A,B), P0 (C,D) and P30 (E,F) for detection of Smarcc1 (A,C,E) and Smarcc2 (B,D,F). Lower panels are the magnifications of the RPE, ONH and retina. DAPI was used for nuclear counterstaining. (G-L′) The recombination pattern mediated by *Dct-Cre* monitored by antibody labeling for GFP expressed by the *Z/EG* reporter at E9 (G), E10 (H), E11 (I), E12 (J,J′), E14 (K,K′) and P7 (L,L′). Higher magnifications of the boxed regions in L are presented on the right (L′). Transverse sections of the OS are shown in J′ and K′. CB, ciliary body; GCL, ganglion cell layer; INL, inner nuclear layer; L, lens; NBL, neuroblast layer; ON, optic nerve; ONH, optic nerve head; ONL, outer nuclear layer; OS, optic stalk; RPE, retinal pigmented epithelium. Scale bars: 100 μm (A,C,E); 25 μm (G-L′; magnifications of A-F).

The two subunits presented a partly overlapping pattern of expression in the optic cup derivatives. At embryonic day (E) 14 and postnatal day (P) 0 both subunits were detected in the cells of the OS and in the RPE progenitors (Fig. 1A-D). Within the developing retina, at E14 and P0, Smarcc1 was detected in the retinal progenitor cells in the neuroblastic layer, while Smarcc2 was mostly in the inner nuclear layer, which is populated by the post-mitotic retinal precursors of ganglion, amacrine and horizontal retinal cells (Fig. 1A-D). Both subunits were detected in the ciliary body (CB) and iris progenitors at the distal tips of the optic cup (Fig. S1).

In the mature retina (P30), the expression of Smarcc1 was reduced below detection in the optic cup/stalk lineages, whereas Smarcc2 was detected in the mature cell types that are derived from the optic vesicle: optic nerve astrocytes, RPE, retinal cell types, CB and iris (Fig. 1E,F, Fig. S1).

The detection of Smarcc2 but not Smarcc1 in differentiated derivatives of the optic cup corresponds with the stage-specific role attributed to each of these subunits, i.e. Smarcc1 in proliferating multipotent progenitors and Smarcc2 in differentiating and mature cell types (Bachmann et al., 2016; Bi-Lin et al., 2021; Zhang et al., 2021).

### Conditional deletion of Smarcc1 or Smarcc2 in the pigmented lineages of the eye using *Dct-Cre* includes the RPE and OS

To examine the Smarcc1- and Smarcc2-specific roles and their possible functional redundancy in the developing and mature pigmented lineages of the eye, we selectively deleted one subunit at a time using the *Dct-Cre* (Davis et al., 2009). To trace the trajectory of Cre-expressing cells, we included the *Z/EG* reporter, in which eGFP is activated in cells where Cre recombinase was active (Novak et al., 2000) (Fig. 1G-L). Detailed analysis of eGFP in the *Dct-Cre;Z/EG* embryos revealed that, in addition to the documented activity of the *Dct-Cre* in pRPE in the dorsal optic cup (Davis et al., 2009), there is notable Cre activity in the developing OS. This expression was evident during OS elongation in the dorsal side, which continued with the pRPE (E12; Fig. 1J,J′) and gradually expanded ventrally as the optic fissure closed (E14; Fig. 1K,K′). By E14, after the closure of the optic fissure, we detected eGFP in the entire RPE, in the ciliary margin zone and in OS-derived astrocyte progenitors surrounding the RGC axons (Fig. 1K). At P7, eGFP was detected in the RPE, pigmented cells of the CB, iris, ON astrocytes and some of the retinal astrocytes that likely migrate from the ONH into the ganglion cell layer (GCL) (Fig. 1L,L′). Thus, *Dct-Cre* deletes the floxed genes in both pigmented and OS lineages.

### Smarcc1 functionally replaces Smarcc2 in the pigmented and OS lineages of the eye

The prominent expression of Smarcc2 in the RPE and ON astrocytes in the adult (Fig. 1F) inferred a key role for this subunit in their maturation and/or maintenance. We therefore examined the phenotype of *Smarcc2^loxP/loxP^;Dct-Cre* (termed *Smarcc2-DCT-cKO*) adult mouse eyes compared to control litter mates.

**Fig. 2.**
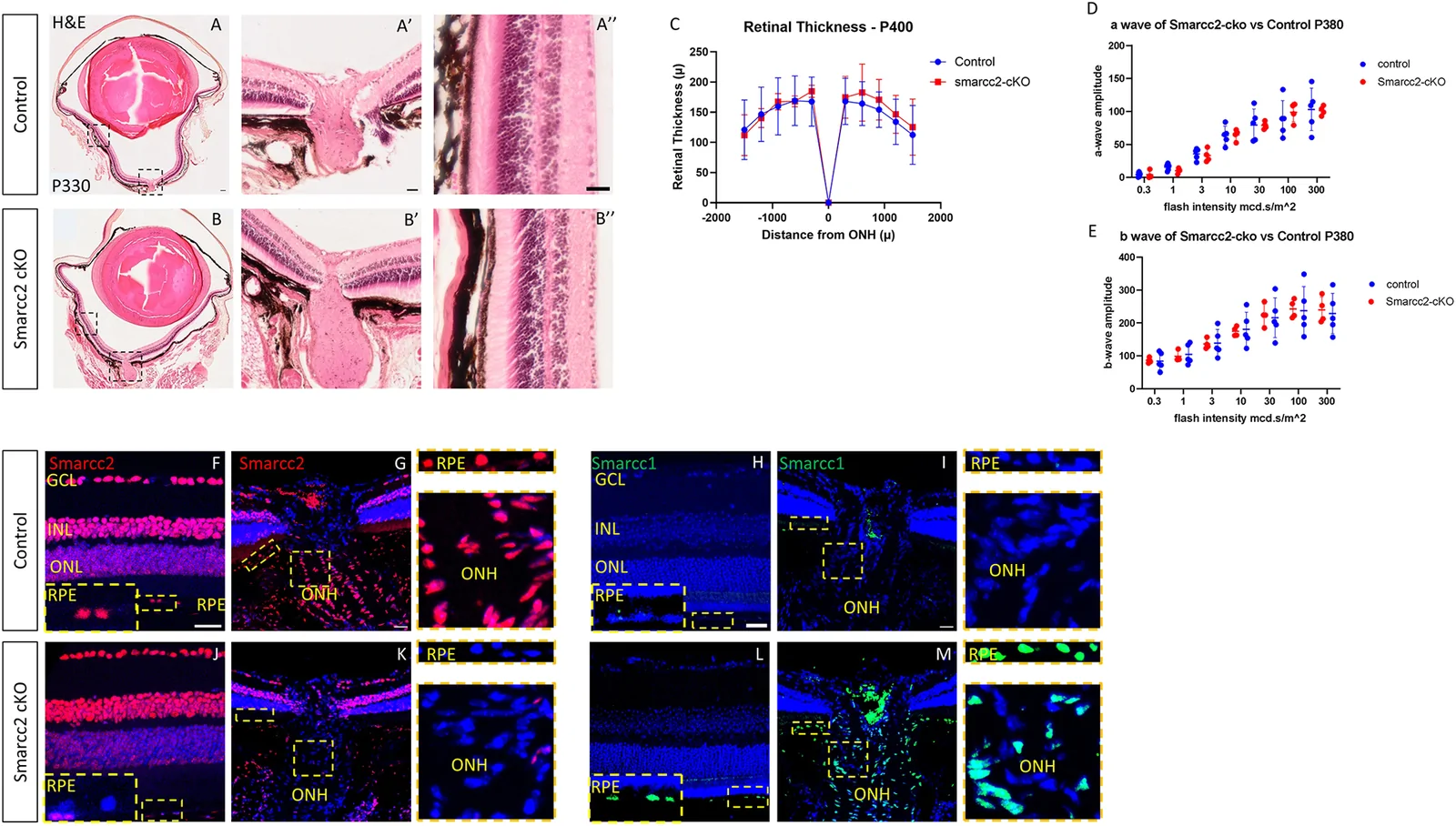
**Smarcc1 functionally replaces Smarcc2 in the pigmented and OS lineages of the eye.** (A-B″) H&E staining of control (A-A″) and Smarcc2-cKO (B-B″) aged mice (P330), with higher magnifications of the boxed areas shown in A′,A″ and B′,B″. (C) Retinal thickness measurements from control and Smarcc2-cKO mice (*n*=3); five points per retinal side were sampled at 300-μm intervals. Error bars represent s.d. (D,E) ERG of aged mice (P380; *n*=4) showing scotopic a-wave (D) and b-wave (E) amplitudes. Error bars represent s.d. The graphs in D,E are reproduced in Fig. S2C,F. (F-M) Immunolabeling of control (F-I) and Smarcc2-cKO (J-M) retinas (P380) for Smarcc2 (F,G,J,K) and Smarcc1 (H,I,L,M). DAPI was used for nuclear counterstaining. Higher magnifications of the boxed areas are shown on the right. GCL, ganglion cell layer; INL, inner nuclear layer; ONH, optic nerve head; ONL, outer nuclear layer; RPE, retinal pigmented epithelium. Scale bars: 100 μm (A,B); 25 μm (A′,A″ B′,B″, F-I).

Histological analysis of aged mice (P330) by Hematoxylin and Eosin (H&E) staining did not reveal any morphological differences in the OC derivatives – RPE, retina, optic nerve, CB and iris – in *Smarcc2-DCT-cKO* compared to control litter mates (Fig. 2A-B″). Although gross morphology and retinal thickness appeared to be preserved in *Smarcc2-DCT-cKO* mice (Fig. 2C), more subtle alterations may result in reduced retinal function with age. However, electroretinography (ERG) analysis at P130, P260 and P380 revealed no significant differences in A- and B-wave amplitudes between control and *Smarcc2-DCT-cKO* mice in both scotopic and photopic conditions (Fig. 2D,E, Fig. S2), indicating maintained retinal function despite Smarcc2 deletion in pigmented and OS lineages.

The lack of detectable phenotype in the *Smarcc2-DCT-cKO* mice suggests functional compensation by Smarcc1. We examined the expression of both Smarcc1 and Smarcc2 comparing the control to *Smarcc2-DCT-cKO*, by antibody labeling (P380). This analysis confirmed the selective expression of Smarcc2 but not Smarcc1 in the control RPE, retina and ONH (Fig. 2F-I) and its selective deletion from the RPE and OS in *Smarcc2-DCT-cKO* eyes (Fig. 2J,K). The analysis further revealed an elevation of Smarcc1 protein in the RPE and ONH, where Smarcc2 was lost due to *Dct-Cre* activity (Fig. 2L,M). The observed elevation of Smarcc1 correlates with its capacity to functionally compensate for the loss of Smarcc2 in these cell types.

### Smarcc1 is required for optic nerve-head development but is redundant with Smarcc2 in the pigmented epithelium of the eye

**Fig. 3.**
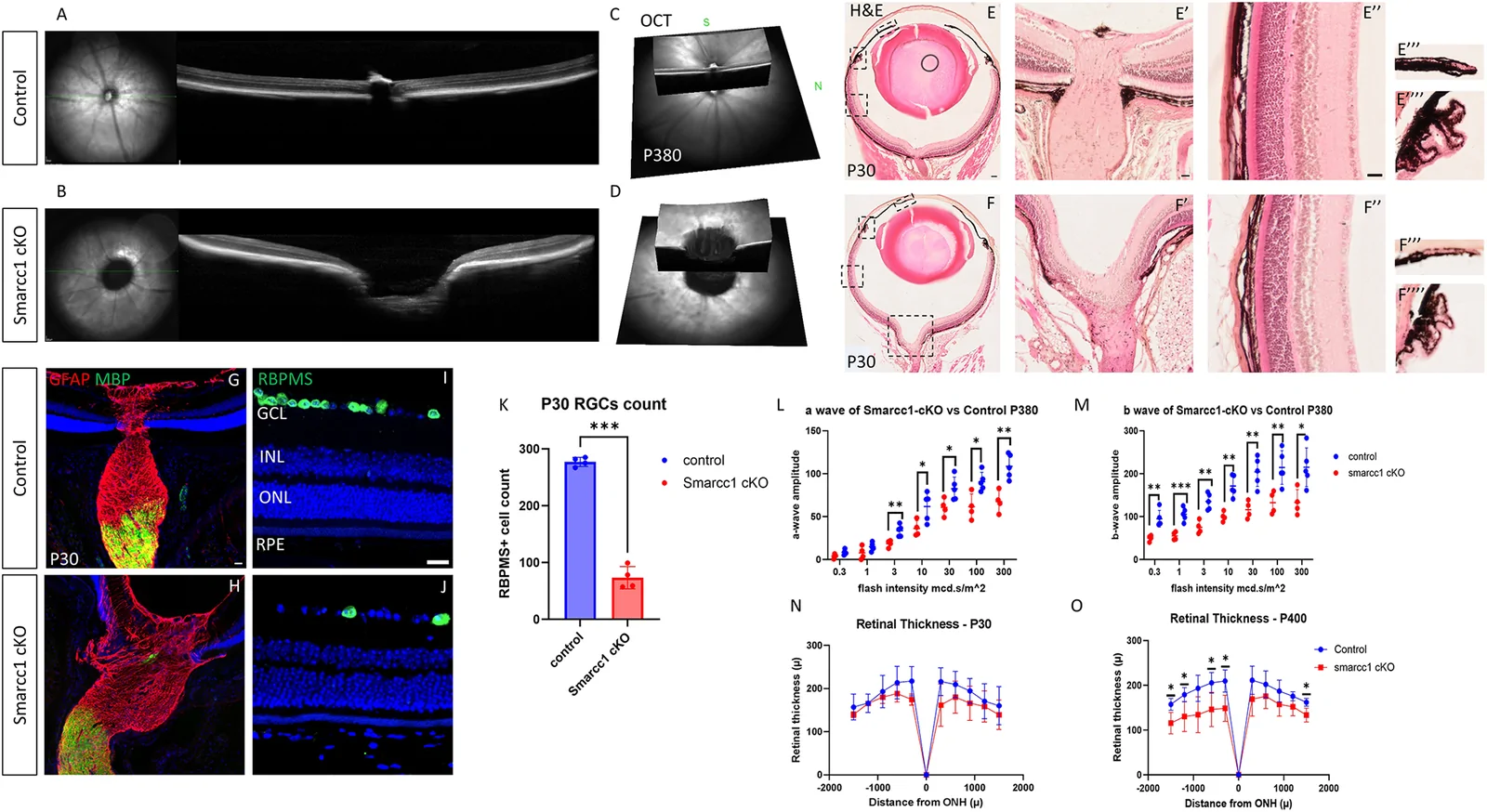
**Smarcc1 is required for ONH development but functions redundantly with Smarcc2 in the pigmented epithelium of the eye.** (A-D) Optical coherence tomography (OCT) imaging of control and *Smarcc1-DCT-cKO* retinas at P380. Representative infrared and B-scan images of control (A) and *Smarcc1-DCT-cKO* (B) retinas. OCT volumetric 3D rendering of ONH area in control (C; S, superior; N, nasal) and *Smarcc1-DCT-cKO* (D) mice. (E-F⁗) H&E staining of control (E, magnifications in E′-E‴) and *Smarcc1-DCT-cKO* (F, magnifications in F′-F⁗) mice (P30). (G-J) Immunolabeling for glial fibrillary acidic protein (GFAP), myelin basic protein (MBP) (G,H), and the RGC marker RBPMS (I,J) in P30 control (G,I) and *Smarcc1-DCT-cKO* (H,J) mice. DAPI was used for nuclear counterstaining. (K) Quantification of RBPMS^+^ RGCs at P30 (*n*=4). (L,M) ERG responses from aged (P380; *n*=4-5) control and *Smarcc1-DCT-cKO* mice show a-wave (L) and b-wave (M) amplitudes under scotopic conditions. The graphs in L,M are reproduced in Fig. S4C,F. (N,O) Retinal thickness measurements of P30 (N) and P400 (O) control and *Smarcc1-DCT-cKO* (*n*=3), were obtained from five points on each retinal side at 300-μm intervals. Error bars represent s.d. *P<0.05, **P<0.01, ***P<0.001 (paired, two-tailed *t*-test). GCL, ganglion cell layer; INL, inner nuclear layer; ONL, outer nuclear layer; RPE, retinal pigmented epithelium. Scale bars: 100 μm (E); 25 μm (E′-I).

We next examined the consequences of Smarcc1 deletion in the OS and pigmented epithelium lineages, in which Smarcc1 is co-expressed with Smarcc2 during embryogenesis (Fig. 1). *In vivo* optical coherence tomography (OCT; A-D) of control (Fig. 3A,C) and *Smarcc1;loxP/loxP;Dct-Cre* (*Smarcc1-DCT-cKO*; Fig. 3B,D) demonstrated a severe malformation of the ONH in *Smarcc1-DCT-cKO* old mice (P380) including atrophy of the adjacent retina and RPE as well as an abnormally excavated ONH. These malformations were already evident by H&E analysis at P30 (Fig. 3 compare E,E′ control with F,F″ of *Smarcc1-DCT-cKO*), whereas the pigmented lineages of RPE, iris and CB preserved their morphology and pigmentation (Fig. 3E‴,E″″,F‴,F″″). These results suggest that Smarcc2 can compensate for the loss of Smarcc1 in the development and morphology of the RPE, iris and CB but not in ONH development. We further characterize the distribution of astrocytes and myelin in the ONH region of control (Fig. 3G) and *Smarcc1-DCT-cKO* (Fig. 3H) by antibody labeling for the astrocyte marker glial fibrillary acidic protein (GFAP) and myelin basic protein (MBP). We observed a disorganized distribution of GFAP in *Smarcc1-DCT-cKO*, while the separation between the myelinated and unmyelinated zones appears preserved in *Smarcc1-DCT-cKO* mutants, as indicated by MBP staining, which identified myelin surrounding nerve fibers outside the unmyelinated zone in both control and mutant mice at P30 (Fig. 3G,H). These findings suggest that Smarcc1 is required for ONH morphology but not for the formation of an unmyelinated zone.

The altered ONH morphology is likely to impact RGC survival. Consistent with this, we observed a significant reduction in RBPMS-positive RGCs in *Smarcc1-DCT-cKO* compared with the control at P30 (Fig. 3I,J, quantification in 3K). Although this reduction in RBPMS-positive GCL was not evident at P0, we did detect at this stage a significant increase in cleaved caspase 3 (CC3)-mediated apoptosis in the *Smarcc1-DCT-cKO* GCL (Fig. S3E).

ERG analysis supports functional impairment, showing significant reductions in both A- and B-wave amplitudes in aged mice (P380) (Fig. 3L,M). In contrast, at earlier stages, ERG differences were not statistically significant, indicating a progressive decline in retinal function over time (Fig. S4). Furthermore, we detected a reduction in overall retinal thickness in the *Smarcc1-DCT-cKO* mice, which was more pronounced at P380 compared to P30, when the difference was not statistically significant (Fig. 3N,O).

These findings reveal a key role for Smarcc1 in ONH morphology. The age-related reduction in retinal thickness and the progressive decline in retinal function are likely driven primarily, but not exclusively, by ONH abnormalities and surrounding retinal atrophy caused by Smarcc1 deficiency.

### The dorsal OS resembles the pRPE progenitors of the optic cup

The phenotype in *Smarcc1-DCT-cKO* is primarily localized to the ONH, which can be divided into two main regions. The first, termed the optic disc, is located at the site where axons exit the eye. The second, situated posterior to the optic disc (termed pONH), includes the glial lamina (lamina cribrosa in humans), a mesh of connective tissue originating from OS astrocytes that supports the retinal axons as they exit the eye (Sun et al., 2009).

The morphogenesis of the optic fissure closure has been investigated primarily at the level of the optic cup, while the progression and regulation of this process at the level of the OS remain mostly unresolved (Patel and Sowden, 2019). To further investigate the pattern of OS maturation, we characterized, through antibody labeling, the expression of several early-expressed, cell type-specific TFs along the proximal-distal and dorsal-ventral axes.

**Fig. 4.**
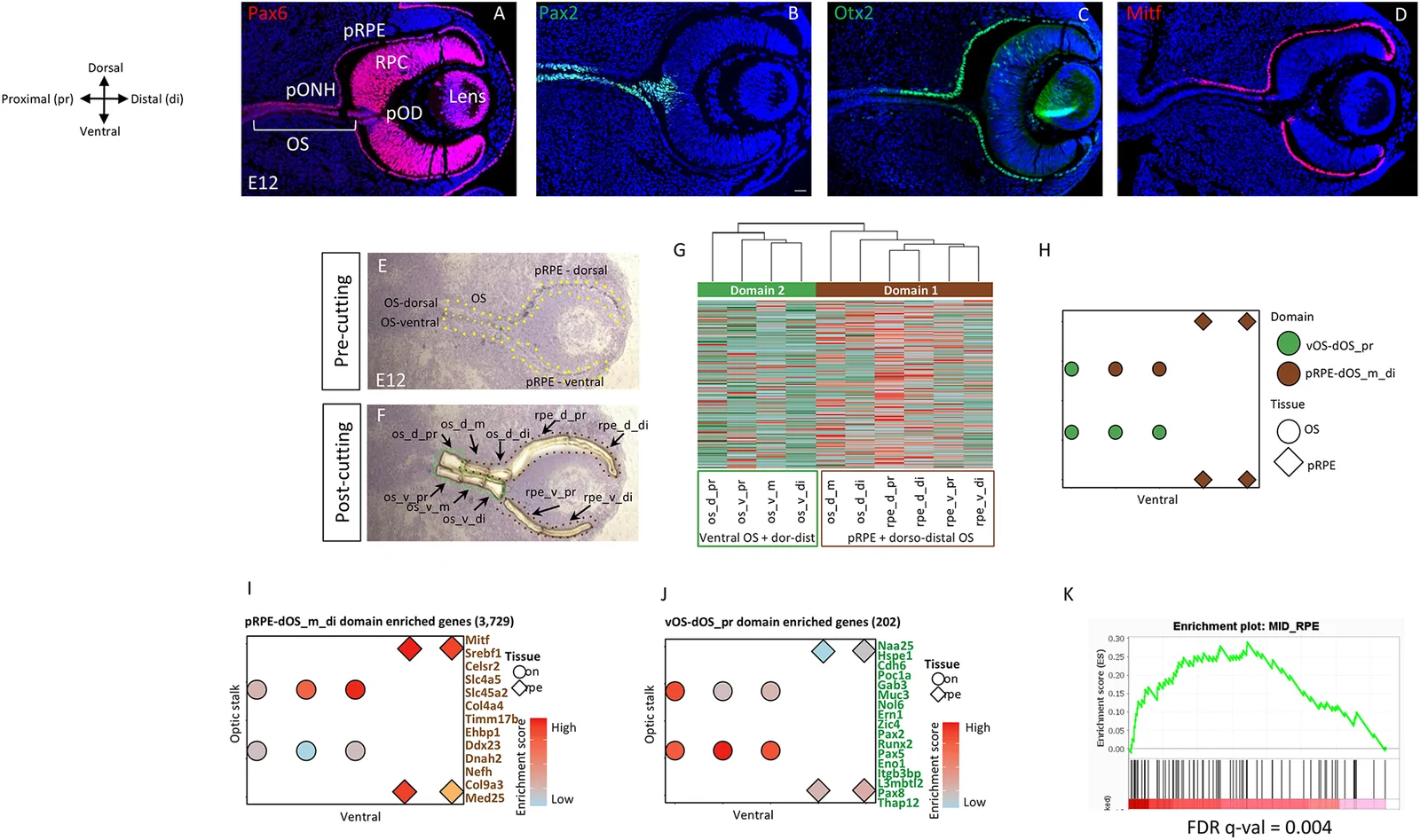
**The dorsal OS continues with the RPE progenitors of the optic cup.** (A-D) Immunolabeling of E12 optic cup sections for Pax6 (A), Pax2 (B), Otx2 (C) and Mitf (D) along the dorsal-ventral (d-v) and proximal-distal (pr-di) axes. DAPI was used for nuclear counterstaining. (E-G) GEO-seq analysis was performed on laser-captured E12 cryosections from the indicated regions before (E) and after (F) sectioning. Whole-transcriptome normalized counts (*n*=3-5 per section) revealed two major transcriptional groups identified by K-means clustering (K=2) and hierarchical clustering (G). (H) Schematic of the sampled regions illustrating two spatial transcriptomic domains: Domain 1 (pRPE-dOS_m_di; brown) and Domain 2 (vOS-dOS_pr; green). (I,J) Representative genes enriched in the pRPE-dOS_m_di domain (I) and genes enriched in the vOS-dOS_pr domain (J). (K) GSEA enrichment for the Mid-RPE gene set (Hu et al., 2019). OS, optic stalk; pOD, prospective optic disc; pONH, prospective optic nerve head; pRPE, presumptive retinal pigment epithelium; RPC, retinal progenitor cells. Scale bar: 25 μm.

Coronal sections presenting the dorsal and ventral OS and cup of control embryos reveal that Pax6 is expressed throughout the early optic cup and also detected, albeit at lower levels, in the OS and the prospective optic disc at E12 (Fig. 4A). At this stage, Otx2 and Mitf are confined to the dorsal layer of the OS that seems to continue with the pRPE of the optic cup, whereas Pax2 expression is restricted to the ventral-proximal OS and the prospective optic disc (Fig. 4B-D).

To characterize the global gene expression profile of the E12 OS and pRPE, we implemented geographical positional sequencing (Geo-seq; Chen et al., 2017) on E12 eyes (Materials and Methods). We sampled three separate sections from each of the dorsal and ventral regions of the E12 OS and two samples each from the dorsal and ventral E12 pRPE (Fig. 4E,F). The spatial transcriptome of E12 OS and pRPE provides with ∼7 million mapped reads, more than 60% mapping ratio, low mitochondrial gene percentage, and 3000 genes per sample on average (Fig. S5A, Table S1). Principal component analysis (PCA) showed that samples primarily separate according to lineage identity (ON versus RPE) and their dorsal-ventral position, indicating that the sample position accounts for major sources of transcriptomic variation (Fig. S5B). To further resolve molecular organization, we applied unsupervised K-means and hierarchical clustering to the full gene-expression matrix. Both approaches consistently identified two transcriptional domains (Fig. 4G and sample schematic in Fig. 4H). Domain 1 (pRPE-dOS_m_di) included all pRPE samples together with the two dorsal-distal OS sections, while Domain 2 (vOS-dOS_pr) comprised all ventral OS samples along with the most proximal dorsal OS section (Fig. 4G,H). Thus, unsupervised clustering uncovered organizational features not captured by PCA, revealing that pRPE and dorsal-distal OS tissues share a distinct transcriptional program.

To define the molecular basis of these domains, we next identified domain-specific signature genes using imputed expression values across all embryos (Table S2; see Materials and Methods). We identified 3729 genes significantly enriched in the pRPE-dOS_m_di domain and 202 genes enriched in the vOS-dOS_pr domain (*P*<0.01; Table S2). Representative domain-specific gene profiles and their enrichment scores across sections are shown in Fig. 4I,J and examples of imputed expression patterns are displayed in Fig. S6.

Gene set enrichment analysis (GSEA) of the pRPE-enriched gene set revealed significant enrichment for pathways associated with forebrain development and light-response programs (Fig. S5E), consistent with the early differentiation stage of these RPE progenitors (Table S3). We next examined pre-defined early (1084 genes), mid (568 genes) and late (567 genes) human RPE gene sets derived from 5-24 weeks of gestation embryos (Hu et al., 2019). The comparative analysis showed strong representation of mid and late RPE gene sets in the pRPE–dOSm_di domain (Fig. 4K, Fig. S5E), further supporting an early RPE differentiation signature in the pRPE and dorsal-distal OS regions.

Thus, consistent with previous observations that the dorsal-distal OS is continuous with the pRPE (reviewed by Bharti et al., 2006; Paşcalău and Badea, 2023), our spatial transcriptomics at E12 show that dorsal-distal OS samples cluster with pRPE and share a broad pRPE-enriched gene signature, supporting the notion that these cells transiently resemble the RPE progenitor-like transcriptional program before astrocyte specification.

### Smarcc1 is required for astrocyte progenitor specification from the dorsal OS

Given the transcriptional differences between dorsal and ventral OS at E12, we next examined how Smarcc1 loss affects OS differentiation and ONH development along the dorsal-ventral axis.

Using transverse sections at the pONH before and after optic fissure closure, we characterized expression of Smarcc1, Smarcc2 and the catalytic subunit Smarca4 in *Smarcc1-DCT-cKO* and control embryos (E12, E14; Fig. S7). In controls, these subunits were detected in both pONH layers at E12 and E14 (Fig. S7A-F). In *Smarcc1-DCT-cKO* embryos, Smarcc1 was absent from the dorsal OS at E12 but retained in the ventral OS (Fig. S7G). By E14, Smarcc1 expression was lost throughout the OS (Fig. S7J). Notably, despite Smarcc1 loss, we did not detect at these stages any change in the expression of Smarcc2 or Smarca4 (Fig. S7H,I,K,L). These results define two important stages for assessing Smarcc1 function: early dorsal OS cells at E12 and astrocyte precursors derived from them at E14.

**Fig. 5.**
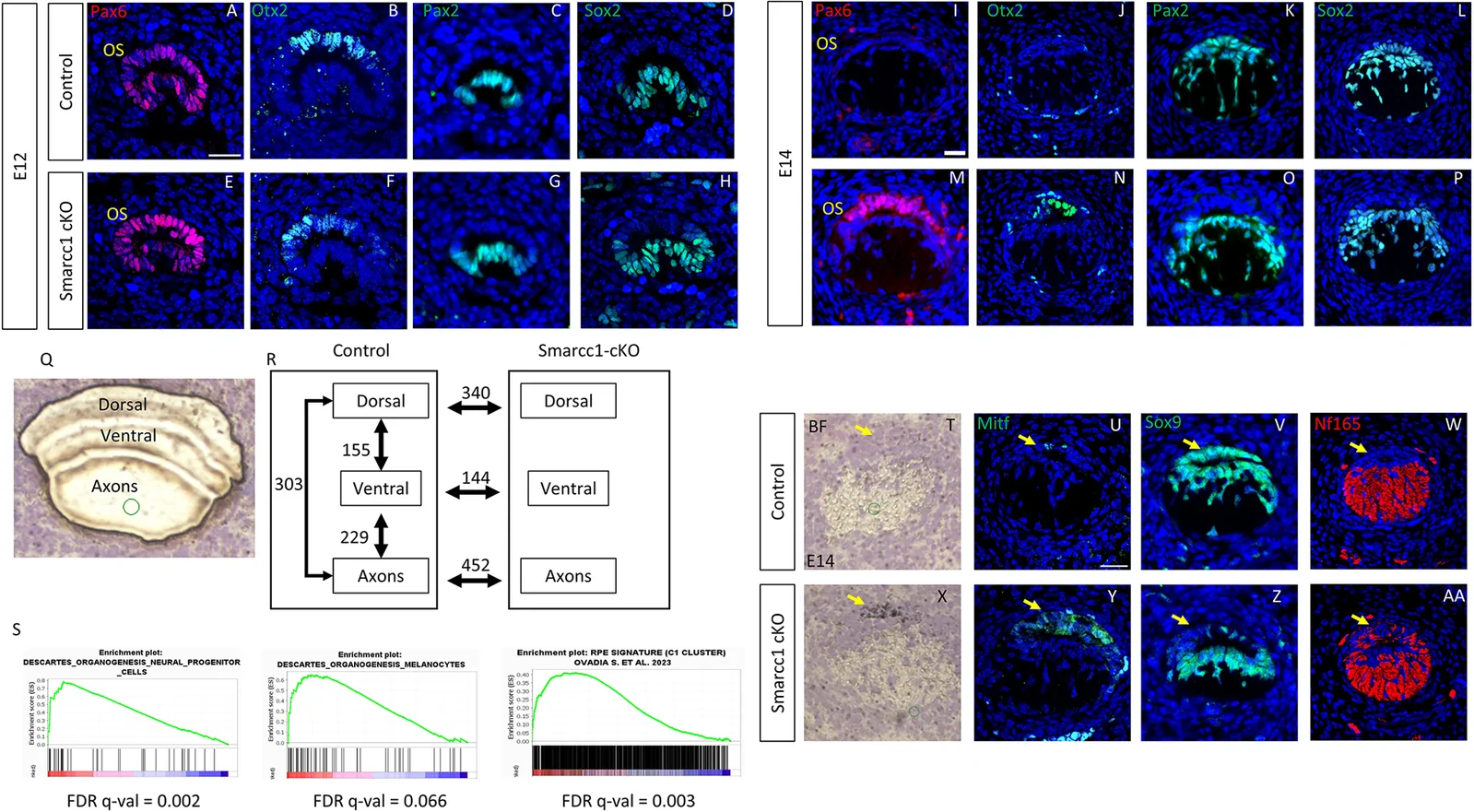
**Smarcc1 is required for dorsal OS specification into astrocyte progenitors.** (A-P) Cross-sections of the OS from E12 (A-H) and E14 (I-P) mouse embryos labeled for Pax6 (A,E,I,M), Otx2 (B,F,J,N), Pax2 (C,G,K,O) and Sox2 (D,H,L,P). (Q) An E14 OS section showing the three regions collected by laser-capture microdissection (LCM; *n*=3-5 samples per region). (R) Schematic summarizing the number of differentially expressed genes (DEGs) between regions and genotypes. (S) GSEA analysis demonstrates that the Smarcc1-cKO dorsal region is enriched for neural progenitor, melanocyte, and mouse E14 RPE signature gene sets. (T-AA) E14 OS cross-sections of control (T-W) and *Smarcc1-DCT-cKO* (X-AA) embryos showing dorsal pigmentation in *Smarcc1-DCT-cKO* (X) but not in control (T), and immunolabeling for Mitf (U,Y), Sox9 (V,Z) and NF165 (W,AA). DAPI was used for nuclear counterstaining. Yellow arrows point to dorsal OS. Panels J and W, as well as M and O, represent different fluorescence channels from the same double-labeled tissue sections. Scale bars: 25 μm.

At E12, prior to optic fissure closure, Pax6 was detected in both dorsal and ventral OS, while Otx2 was restricted to the dorsal OS, consistent with its pRPE identity (Figs 4A,B, 5A,B). In contrast, the astrocyte progenitor TFs Pax2 and Sox2 were confined to the ventral OS (Figs 4D, 5C,D). Despite the reduction of Smarcc1 in the dorsal OS of *Smarcc1-DCT-cKO* embryos, we did not detect a change in the expression pattern of Pax6 or Otx2 at this stage (Fig. 5E-H), suggesting that at this stage the early dorsal OS identity is retained despite Smarcc1 loss.

By E14, following optic fissure closure, both Pax6 and Otx2 proteins were nearly undetectable in the OS of the control embryos (Fig. 5I,J; the gradual reduction of Otx2 in dorsal OS is presented in Fig. S9). At this stage, astrocyte progenitors begin to delaminate from the dorsal OS and migrate into the forming optic nerve to ensheathe retinal axons (Fig. 5K,L). Accordingly, Pax2 and Sox2 expression expanded to both dorsal and ventral OS layers (Fig. 5K,L).

In contrast, *Smarcc1-DCT-cKO* embryos retained Pax6 and Otx2 expression in the dorsal OS at E14, while Pax2 and Sox2 were absent from this region (Fig. 5M-P). This suggests that the dorsal OS cells fail to initiate differentiation into astrocyte progenitors and instead maintain the early pRPE-like identity.

Together, these findings reveal a progressive differentiation pattern within the OS and the role of Smarcc1 in both stages. At E12, dorsal and ventral OS cells exhibit distinct identities, with dorsal cells showing pRPE-like features. As development proceeds and the fissure closes, this distinction is lost, and all OS cells acquire astrocyte progenitor characteristics, marked by Sox2 and Pax2 expression. Smarcc1 is essential for this transition, particularly for the transition of dorsal OS cells from a pRPE-like state to astrocyte progenitors.

For global characterization of the differences between the dorsal and ventral regions of the OS during embryonic development and to reveal the transcriptional network governed by Smarcc1, we characterized the OS transcriptome at E14 by GEO-seq (Chen et al., 2017). This stage was chosen as the earliest stage a phenotype was detectable, and because the deletion occurs in all OS cells by this stage.

Samples were collected by laser-capture microdissection (LCM) from three regions of the E14 OS: dorsal, ventral, and RGC axons (Fig. 5Q) from four control and five *Smarcc1-DCT-cKO* embryos, enabling a statistical evaluation of the variations between and within the samples.

From each sample we obtained, using Smart-Seq2, a mean depth of 30 million sequenced reads per sample. The raw reads were mapped onto the mouse reference genome (mm10) and an average of about 12,000 unique genes were identified per sample (Fig. S8A, Table S4).

The PCA presented high correlation between the samples isolated from the same regions (Fig. S8B). Notably, the dorsal samples showed separation in the PCA between the *Smarcc1-DCT-cKO* and the control, while in the ventral and axon regions, control and *Smarcc1-DCT-cKO* samples clustered together.

We determined the differentially expressed genes (DEGs) between the three regions, and between the control and *Smarcc1-DCT-cKO* samples of each region using DESeq2 [|log2(fold change)|>0.5, *P-*adj<0.05] (Love et al., 2014; Fig. 5R, Table S5).

GSEA (Mootha et al., 2003; Subramanian et al., 2005) comparing gene expression between the dorsal *Smarcc1-DCT-cKO* and control dorsal regions revealed an enrichment for gene sets involved in melanocyte and neural progenitor genes (Fig. 5S, Table S6). Furthermore, GSEA of gene sets from E14 mouse pRPE, defined by Geo-seq (Ovadia et al., 2023), identified significant enrichment for pRPE signature genes in the E14 *Smarcc1-DCT-cKO* dorsal OS, but not in the ventral OS or the axon-rich regions (Fig. 5S, Table S6). This enrichment suggests that loss of Smarcc1 arrests dorsal OS cells at an early pRPE-like stage. Consistent with this, at E14 *Smarcc1-DCT-cKO* OS, but not controls, displayed pigmented accumulation and Mitf expression (Fig. 5T,U,X,Y), whereas Sox9, a marker of astrocyte progenitors normally present in dorsal OS in controls (Fig. 5V), was markedly reduced in the *Smarcc1-DCT-*cKO (Fig. 5Z). We further detected NF165 (NEFM), which in the control is confined to the ventral region of the optic nerve, in the dorsal OS of *Smarcc1-DCT-cKO*, suggesting cryptic onset of neural precursor genes (Fig. 5W,AA). Consistent with this, transcriptomic analyses revealed decreased expression of Hes1, together with increased expression of several proneural genes normally repressed by Hes1 (Kageyama et al., 2008), indicating partial derepression of neural gene programs following Smarcc1 loss in OS progenitors.

### Loss of Smarcc1 disrupts astrocyte progenitor proliferation dynamics in the OS

**Fig. 6.**
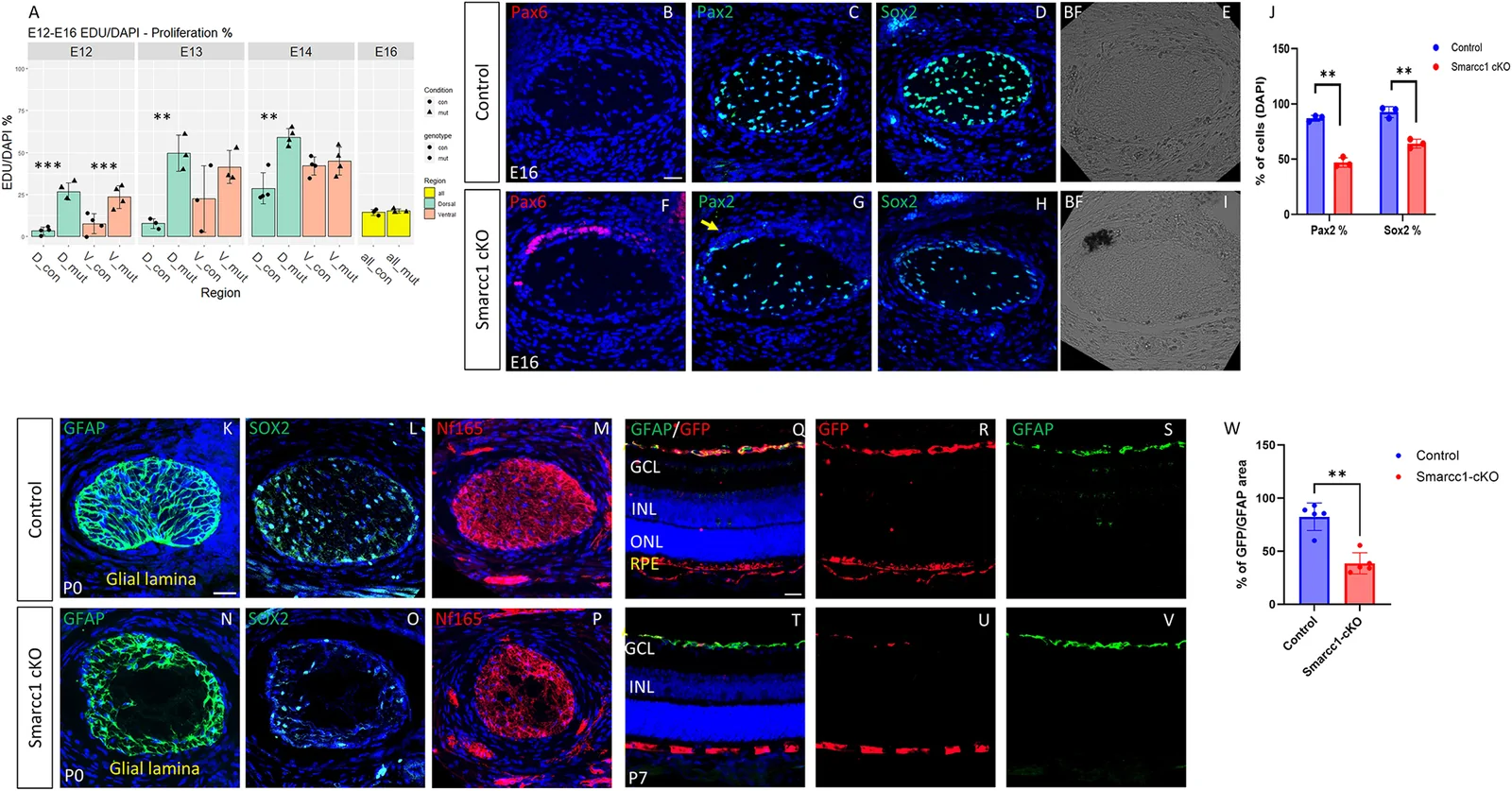
**Smarcc1 is required for astrocyte progenitor proliferation dynamics, formation of the glia lamina and migration into the retina.** (A) Quantification of EdU/DAPI-labeled cells in dorsal and ventral OS regions of control (circles) and *Smarcc1-DCT-cKO* (triangles) embryos following a 3-h pulse-chase at E12, E13, E14 and E16 (in E16, we did not separate between dorsal and ventral domains) (*n*=3-4). (B-I) E16 OS cross-sections of control (B-E) and *Smarcc1-DCT-cKO* (F-I) embryos labeled for Pax6 (B,F), Pax2 (C,G) and Sox2 (D,H), as well as bright-field images (E,I). (J) E16 OS Pax2 and Sox2 quantification (marker/DAPI) (*n*=3). (K-P) P0 OS cross-sections of control (K-M) and *Smarcc1-DCT-cKO* (N-P) antibody staining for GFAP (K,N), Sox2 (L,O) and NF165 (M,P). (Q-V) P7 retinal sections of control (Q-S) and *Smarcc1-DCT-cKO* (T-V) labeled with GAFP and GFP. Merged channels shown in Q,T and separate channels in R,S,U,V. (W) Quantification of the GFP-positive area out of the GFAP area (GFP/GFAP) showing the percentage of *Dct-Cre* astrocytes in retinas (*n*=5). DAPI was used for nuclear counterstaining. Error bars represent s.d. Scale bars: 25 μm. **P<0.01, ***P<0.001 (paired, two-tailed *t*-test).

The expansion of the dorsal OS in *Smarcc1-DCT-cKO* pONH may result from altered cell proliferation, a process known to be regulated by SWI/SNF complexes in different lineages, including the RPE (Braun et al., 2021; Elbaz-Hayoun et al., 2023; Ovadia et al., 2023). To investigate this, we assessed cell proliferation using a 5-ethynyl-2′-deoxyuridine (EdU) pulse-chase assay (3-h pulse; Salic and Mitchison, 2008), quantifying EdU incorporation in both dorsal and ventral OS cells (Fig. 6A, Fig. S10).

In control mice, the average EdU uptake in the dorsal OS was approximately 5% of cells, at both E12 (3.25±2.3%; mean±s.d.) and E13 (7.6±3%), with a marked increase observed at E14 (28.6±10%). In contrast, the ventral region, where the migrating astrocyte progenitors are located, showed a gradual increase in EdU-positive cells: 7.6±6% at E12, 22±19% at E13 and 42±5.3% at E14 (Fig. 6A).

In contrast, in the *Smarcc1-DCT-cKO* mice, the dorsal OS exhibited a significantly higher proportion of EdU-positive cells as early as E12 (26.7±5.2%), increasing to 49±10.8% at E13 and 59±5.16% at E14 (Fig. 6A). Similarly, the ventral region showed accelerated proliferation, with 49.5±8.2% of cells EdU-positive already by E13–1 day earlier than in controls (Fig. 6A).

These findings suggest that, in control embryos, OS cell proliferation is linked to the acquisition of astrocyte progenitor identity. Loss of Smarcc1 disrupts this coordination, leading to premature and excessive proliferation in both dorsal and ventral regions. Consequently, the average number of progenitors in the *Smarcc1-DCT-cKO* pONH was significantly higher compared to the controls at E14 (control: 18.6±4.4 cells; *Smarcc1-DCT-cKO*: 41.5±14.9 cells; Fig. S10).

By E16, the difference in cell proliferation between control and *Smarcc1-DCT-cKO* OS cells was no longer observed. In both control and *Smarcc1-DCT-cKO*, we detected 18±2% and 19±3% cells in S-phase (Fig. 6A). This convergence suggests that the early proliferative surge in the *Smarcc1-DCT-cKO* OS is transient and may be followed by a compensatory slowdown or normalization of cell-cycle activity.

### Smarcc1 is essential for ONH astrocyte differentiation and migration

At E16, the OS in the control embryos lacked Pax6- or pigment-expressing cells and astrocyte progenitors, marked by co-expression of Pax2 and Sox2, appeared evenly distributed around the RGC axons (Fig. 6B-E). In contrast, the dorsal OS of the *Smarcc1-DCT-cKO* contained a small cluster of ectopic pigmented cells and Pax6-positive cells (Fig. 6F,G), likely remnants of dorsal OS cells that failed to acquire astrocyte progenitor identity due to Smarcc1 loss. Despite this, the total number of cells within the *Smarcc1-DCT-cKO* OS was comparable to that in the controls (82±9 in control, 89±18 in *Smarcc1-DCT-cKO*; mean±s.d.; Fig. S10), but these cells were mostly confined to the OS periphery. Moreover, the proportions of Sox2 and Pax2 in these cells were significantly reduced (Pax2: control 86±3%, *Smarcc1-DCT-cKO* 46±3%; Sox2: control 92±3%, *Smarcc1-DCT-cKO* 65±3%; Fig. 6H-J). Thus, Smarcc1 loss leads to diminished expression of astrocyte progenitors TFs and impaired migration into the developing optic nerve, indicating failure in astrocyte progenitor maturation.

ONH astrocytes play a crucial role in maintaining structural integrity for RGC axons exiting the eye by forming the glial lamina, a dense astrocytic meshwork that ensheathes RGC bundles in the unmyelinated prelaminar pONH. In controls at P0, this meshwork expressed Sox2 and GFAP and surrounded retinal axons labeled by NF165 (Fig. 6K-M). In *Smarcc1-DCT-cKO* mice, however, astrocytes failed to migrate into the pONH, remained at the periphery, and exhibited reduced Sox2 expression (Fig. 6N,O). Correspondingly, RGC axons were depleted in astrocyte-deficient regions (Fig. 6P), consistent with RGC death observed at this stage (Fig. S3). These findings highlight the essential role of Smarcc1 in astrocyte differentiation, glial lamina formation, ONH integrity and postnatal RGC survival.

To further assess the role of Smarcc1 in ONH astrocyte migration into the retina, we traced astrocytes in which *DCT-Cre* was active by utilizing the Z/EG reporter (Novak et al., 2000; Fig. 6Q-W, Fig. S11). This analysis revealed that the *Dct-Cre* transgene was active in some, but not all, Pax2-positive optic discs in both control and *Smarcc1-DCT-cKO* embryonic eyes (E14.5; Fig. S11A,B). In P7 control (*Dct-Cre*; Z/EG) astrocytes, eGFP was detected in approximately 82±11.5% (mean±s.d.) of retinal astrocytes, detected by GFAP, in the RGC layer, indicating their origin in the ONH (Fig. 6Q-S). In *Smarcc1-DCT-cKO* P7 mice, the proportion of eGFP to GFAP in the inner retina was significantly reduced to ∼38±8.8% (Fig. 6T-W), suggesting impaired astrocyte migration from the ONH into the retina (see scheme in Fig. S11C-E).

Together, these results demonstrate that Smarcc1 is required for the proper development, organization and migratory capacity of ONH astrocytes. Its loss disrupts glial lamina formation and astrocyte positioning, compromising ONH integrity and leading to RGC degeneration.

## DISCUSSION

In this study, we uncover a spatiotemporal mechanism that governs astrocyte specification and differentiation in the OS and identify Smarcc1 as a key chromatin regulator of these processes. Using spatial transcriptomics integrated with genetic loss-of-function analysis, we demonstrate that the dorsal OS transiently adopts a pigmented, RPE-like transcriptional identity, whereas the ventral stalk initiates Pax2/Sox2-positive astrocyte progenitor specification earlier, generating a ventral-to-dorsal wave of differentiation that coincides with optic fissure closure and optic nerve morphogenesis. Although Smarcc1 and Smarcc2 act redundantly in the RPE and other pigmented lineages, we find that Smarcc1 alone is required to suppress the RPE-like program in dorsal stalk progenitors and to regulate their proliferation rate, subsequent migration, and contribution to glial-lamina formation, a specialized structure that provides lifelong support for RGC axons. Thus, Smarcc1 performs a unique, non-redundant role in OS-derived astrocyte specification and ONH development.

### Spatial and temporal organization of astrocyte progenitor specification in the OS

Our findings support a dorsal-ventral organization of astrocyte progenitor specification within the OS, complementing the well-established proximal-distal patterning of the optic vesicle into OS, RPE, and neural retina lineages. This spatial organization is coordinated by positional cues from adjacent tissues (reviewed by Bharti et al., 2006; Fuhrmann et al., 2014; Tao and Zhang, 2014; Moreno-Marmol et al., 2018).

**Fig. 7.**
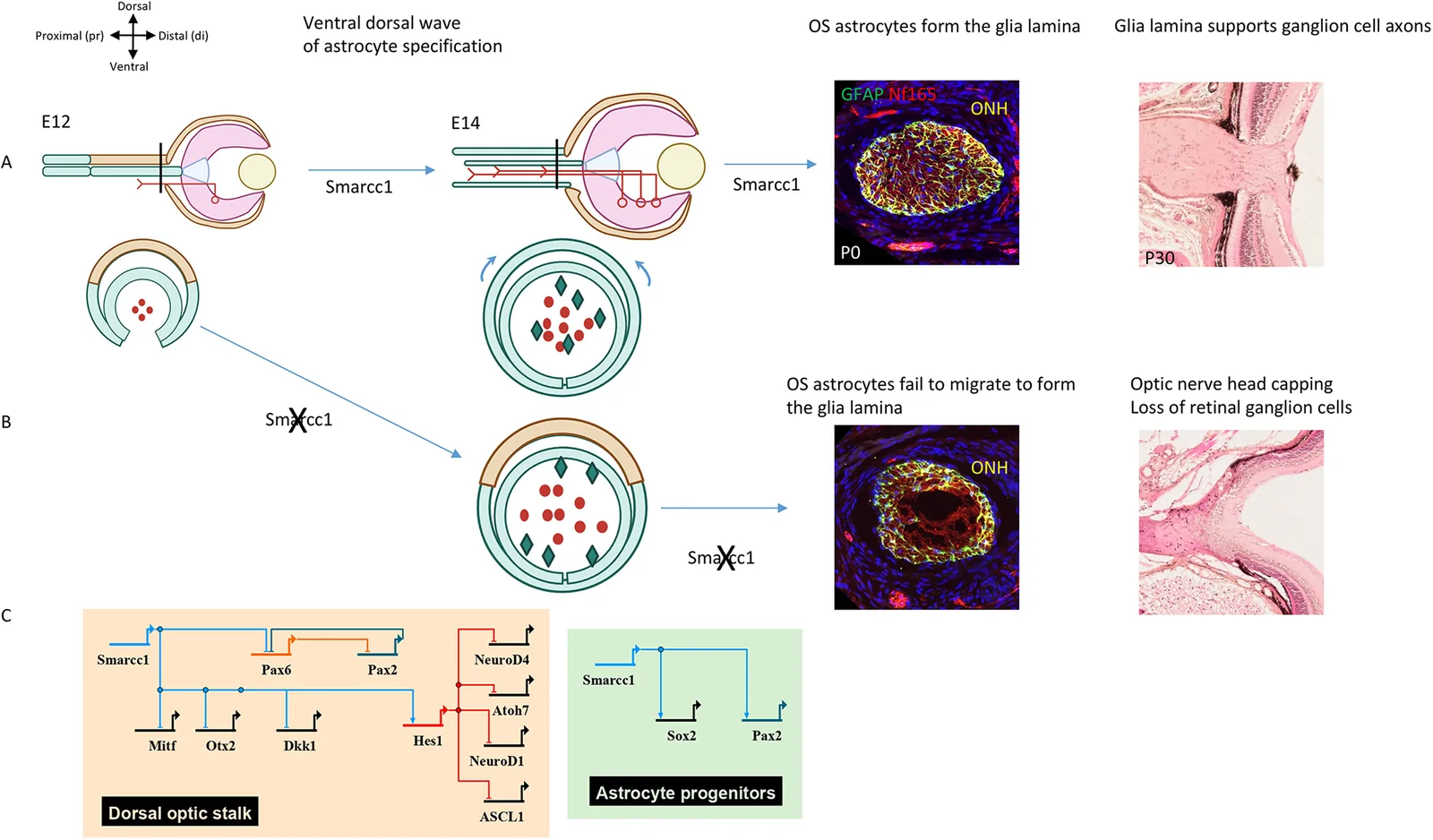
**Summary model of Smarcc1-dependent OS development, astrocyte lineage progression, and regulatory programs revealed by transcriptomics.** (A,B) Schematic summarizing the developmental trajectory of OS-derived astrocytes in control versus Smarcc1-cKO embryos. In control optic nerve development (A), Smarcc1 promotes dorsal OS identity at E12-E14, establishing the dorsal-to-ventral wave of astrocyte progenitor specification (arrows show the direction of differentiation). The pale/gray arc shape in A represent the optic disc, which also contain a pool of retinal astrocytes and is located around the exit point of the RGC axons (marked in red). The astrocyte progenitors are represented by green diamonds. By P0, these progenitors migrate into the ONH and assemble the glial lamina as shown in the middle panel by immunolabeling for GFAP (astrocytes) and NF165 (axons) in a P0 ONH section (nuclei labeled with DAPI). The labeling for NF165 in A and for GFAP in B is the same as that in Fig. 6M and Fig. 6N, respectively. The mature glial lamina (P30) supports RGC axons, ensuring long-term RGC survival through life. The corresponding ONH architecture is shown in the panel on the right using H&E staining. In Smarcc1-cKO embryos (B), loss of Smarcc1 disrupts dorsal OS patterning, impairs astrocyte progenitor specification, blocks their migration into the ONH, and prevents glial lamina formation, ultimately leading to loss of axons, ONH cupping and RGC degeneration. The H&E images in A and B are the same as those shown in Fig. 3Eʹ,Fʹ. Created in BioRender by Ashery Padan, R., 2026. https://BioRender.com/egpk9yw. This figure was sublicensed under CC-BY 4.0 terms. (C) Diagram highlighting the key gene regulatory networks identified through our transcriptomic analysis (illustrated by BioTapestry; Longabaugh et al., 2005). These networks include factors that regulate the early transition from a pRPE-like transcriptional state to astrocyte progenitors. At this stage, Smarcc1 is required to downregulate pigmented epithelium determinants (Pax6, Otx2, Mitf) and for the inhibition of proneural determinants possibly through Hes1 regulation. Smarrc1 is required (directly or indirectly) for activation of the astrocyte TFs (Pax2, Sox2, Sox9). During astrocyte differentiation, Smarcc1 is required for astrocyte identity, including for Sox2 expression. Together, these results outline a Smarcc1-dependent molecular program controlling the emergence, specification, and functional maturation of OS-derived astrocytes.

Previous studies have noted that dorsal OS cells exhibit RPE-like characteristics (Silver and Sapiro, 1981; Bharti et al., 2006; Paşcalău and Badea, 2023). Our spatial transcriptomic analysis extends these observations by demonstrating at a genome-wide level that E12 dorsal-distal OS samples cluster with pRPE and display a broad RPE-enriched transcriptional signature. This establishes a dorsal RPE-like identity within the OS and provides systems-level support for continuity between these domains (Fig. 7A). The transcriptional similarity may reflect a shared lineage trajectory, possibly due to transient epithelial cell rearrangements and cell stretching occurring during optic cup morphogenesis, processes documented in teleost embryos but not yet demonstrated in mammals (Kwan et al., 2012; Heermann et al., 2015; Eckert et al., 2019; Moreno-Marmol et al., 2021). Alternatively, the similarity in gene expression may arise from shared exposure to local inductive cues (reviewed by Morcillo et al., 2006) that activate similar gene regulatory networks independent of lineage origin. Definitive resolution of these relationships will require lineage-tracing and live-imaging approaches to distinguish between lineage continuity and inductive convergence during OS formation.

The distinct molecular properties of the dorsal and ventral OS help explain the phenotypes observed in Pax6 and Vax1/2 misexpression and functional studies, in which disruption of RPE-program repression leads to pigment accumulation or expansion of retinal/RPE fates at the expense of OS identity (Torres et al., 1996; Hallonet et al., 1999; Schwarz et al., 2000; Mui et al., 2005). These findings underscore the differential molecular properties of the dorsal and ventral OS and their distinct responses to genetic perturbations.

Notably the ventral to dorsal wave of astrocyte specification is tightly coupled with two key developmental events: optic fissure closure (completed by E12.5) and axonal extension from RGCs toward the brain. The dorsal stalk is the last to differentiate into astrocyte progenitors, indicating a spatially coordinated maturation process (Chan et al., 2020). In this context, the inner layer of the OS, which interfaces with extending axons, appears to proliferate more and delaminate prior to the outer layer, potentially regulating both the timing and quantity of astrocyte production. This layered maturation may reflect a mechanism to ensure that astrocyte differentiation is synchronized with axonal pathfinding and optic nerve formation (Huxlin et al., 1992; Tao and Zhang, 2014). Such coordination is crucial, as astrocytes not only support axonal growth but also contribute to the formation of the gliovascular network and optic nerve architecture.

Our data further indicate that the posterior ONH region is delayed in acquiring astrocytic identity and instead retains RPE-like transcriptional features after the adjacent OS regions have committed to the astrocyte lineage. This conclusion is supported by the expression patterns of Pax6, Mitf and Otx2 (Figs 4, 7A,B, Fig. S9), suggesting that the posterior ONH may represent a transitional or regulatory zone, where cell fate decisions are temporally postponed, possibly to coordinate with late-stage events such as optic cup morphogenesis, vascular invasion, and glial lamina formation as well as postnatal growth at the posterior ONH (Bernstein et al., 2020). Thus, the ONH exhibits a delayed onset of astrocyte marker expression relative to the rest of the OS and retains pRPE-like signatures late into development, indicating unique temporal control of fate specification aligned with its specialized role in optic nerve formation and function.

### Functional redundancy and divergence of Smarcc1 and Smarcc2 in eye development

Within this spatial framework, our data highlight distinct roles for Smarcc1 and Smarcc2, the core components of the SWI/SNF complex. In Smarcc2-deficient models, we observe upregulation of Smarcc1, with no apparent disruption in the morphology or function of the RPE, OS, iris or CB. This suggests that Smarcc1 is sufficient to compensate for Smarcc2 loss, consistent with previous studies indicating functional redundancy between these subunits in maintaining BAF complex functions (Fig. 2; Narayanan et al., 2018). The expression of Smarcc2 in the pigmented lineages of the eye is nevertheless important for compensating for Smarcc1 loss in the RPE, iris and CB lineages as these structures seem morphologically intact in the *Smarcc1-DCT-cKO* (Fig. 3). Thus, pigmented lineages derived from the outer optic cup seem to require either Smarcc1 or Smarcc2, which together ensure robust differentiation of these tissues.

### Smarcc1 is obligatory for OS patterning and for OS astrocyte functions

Nevertheless, the loss of Smarcc1 does expose its non-redundant role in ONH development, a pivotal structure for retinal function. Upon Smarcc1 conditional inactivation, we detected ONH dysgenesis resulting in capping of the optic nerve head. The alterations in the ONH seem to result in non-cell-autonomous effect on the retina, as it was accompanied by early loss of ganglion cells and reduced retinal activity based on ERG analysis in aged mice. Nonetheless, future studies using a Cre driver restricted to the RPE, but not the OS, will be necessary to determine whether Smarcc1 expression in the embryonic RPE contributes to RPE differentiation and maintained functions in the adult.

Detailed characterization of *Dct-Cre* activity onset shows that Smarcc1 deletion begins at E12 in the dorsal OS, prior to astrocyte specification, and expands ventrally by E14, affecting cells already committed to the astrocyte lineage. Phenotypic analysis indicates that Smarcc1 is essential at both stages. First, it is required for the specification of dorsal OS cells into astrocytes. In its absence, these cells fail to initiate astrocyte differentiation and instead retain progenitor-like, RPE-like characteristics, marked by elevated proliferation and expression of Mitf, Otx2 and Pax6, and failure to upregulate Pax2 (Fig. 7B). Although dorsal cells initially expand due to increased proliferation, this is not sustained, and the pigmented cell patch observed at P0 is small and does not integrate into the ONH structure. We further detected ectopic NF165 expression in the dorsal OS. Consistent with this, RNA-seq analysis revealed reduced Hes1 expression and elevated expression of several proneuronal genes in the Smarcc1 mutant OS. Hes1 is a Notch/Hedgehog-responsive transcriptional repressor documented to inhibit proneural genes and to contribute to OS and optic cup boundary maintenance (Kageyama et al., 2008; Wall et al., 2009; Bosze et al., 2020). This finding therefore supports a model in which Smarcc1 plays a role in repression of neuroepithelial identity, at least partly through Hes1, and that this contributes to the timely acquisition of glial fate (Figs 5AA, 7B).

In contrast, ventral OS cells in the *Smarcc1-DCT-cKO* model do acquire astrocyte progenitor identity before Smarcc1 deletion occurs and thus provide an opportunity to study Smarcc1 loss during astrocyte maturation. By E16, the distribution of OS cells originating from the ventral side seems altered, with more cells at the periphery of the OS and fewer cells delaminated between the RGC axons. By P0, these astrocyte progenitors populate mostly the periphery of the optic nerve and at this stage we detect regions that are devoid of axons, based on NF165 distribution (Fig. 6). Furthermore, *Smarcc1-cKO* astrocytes seem to fail to migrate into the nerve fiber layer of the retina. These results show that Smarcc1 is not only obligatory for OS cells to gain astrocyte identity, but also for normal astrocyte differentiation with respect to migration into the optic nerve and to the retina. The detected reduction in Sox2 already at E16 may explain some of these phenotypes as Sox2 is required for astrocyte differentiation in the retina and it functions in a unique subset of ONH astrocytes for postnatal optic nerve growth (Kautzman et al., 2018; Bernstein et al., 2020; Wang et al., 2022). The transcriptomic analysis of the *Smarcc1-DCT-cKO* further revealed a reduction in expression of ECM modulators such as Mmp14 and Adam15, which have been shown to control cell migration (Martin et al., 2002; Garmon et al., 2018). Collectively, Smarcc1 appears to act upstream of multiple pathways contributing to astrocyte identity, proliferation and migration properties. These multifaceted roles of Smarcc1 establish its essential function in ONH development, with direct relevance to optic nerve pathologies. Smarcc1 mutant ONH defects parallel OS patterning failures in Vax1/2 models (Morcillo et al., 2006) and developmental failure of ONH astrogenesis in Sox2 conditional mutants, in which impaired astrogenesis disrupts glial lamina formation and RGC axon support (Bernstein et al., 2020). Astrocyte dysfunction in the adult optic nerve also contributes to glaucoma models and post-injury scenarios, in which ONH astroglial disturbances exacerbate RGC loss (Lee et al., 2025). Emerging work identifying distinct ONH astrocyte subtypes and exploring their targeted manipulation in glaucoma further highlights the need to understand the developmental origins and molecular regulation of these glial populations (Cameron et al., 2024). Thus, elucidating the developmental mechanisms governing ONH astrocyte formation is essential for understanding optic nerve dysfunction and for informing future neuroprotective strategies.

### Smarcc1 loss in retinal astrocytes is compensated for by normal cells

The formation of the astrocyte template and the regulation of astrocyte numbers in the retinal nerve fiber layer is a tightly controlled developmental process, crucial for ensuring proper retinal vasculature patterning and integrity (Paisley and Kay, 2021). During development, an excess of astrocytes is produced, far greater than the number present in the mature retina. Notably, recent studies show that microglia play an active role in this regulation by selectively removing surplus astrocytes through non-apoptotic mechanisms, a process essential for normal retinal vascular development (Punal et al., 2019). In our study, we observed that Smarcc1 loss reduces the number of mutant astrocytes, but total astrocyte counts in the retina remain unchanged. This suggests that genotypically normal astrocytes – likely sourced from the retinal disc – can compensate for the loss of a mutant subpopulation. Such compensation demonstrates cellular plasticity and provides direct evidence for robust homeostatic mechanisms that preserve the astrocyte template despite perturbations. These findings underscore the broader principle of glial compensation, which may have relevance not only during development but also in the context of retinal disease, repair or regeneration.

## MATERIALS AND METHODS

### Animals

Mice were kept at the Tel Aviv University Animal House Facility. Animal use was approved by the Tel Aviv University Animal Care and Use Committee (Approval Number 28-02-2021). The analysis was conducted on *Baf155^loxP/loxP^*, *Dct-Cre;Z/EG* or *Baf170^loxP/loxP^;Dct-Cre;Z/EG* and control littermates that do not carry the *Dct-Cre* (the genotypes of the GEO-seq samples are listed in Table S1). The *Baf155^loxP/+^* (Choi et al., 2012), *Baf170^loxP/+^* (Tuoc et al., 2013a), *Dct-Cre* ((Davis et al., 2009); MMRRC ID 071664-UCD) and Z/EG mouse lines ((Novak et al., 2000); IMSR_JAX:004178) were maintained on a C57Bl6/J genetic background. The primers used for genotyping are listed in Table S7.

### ERG

ERG recordings were performed in a dark room following overnight dark adaptation. Recordings were conducted using a portable HMsERG unit (OcuScience). Pupils were dilated using 0.5% tropicamide (Mydramide, Dr. Fischer), and 20 min later animals were sedated by a subcutaneous injection of ketamine and xylazine and placed inside a Faraday cage to minimize noise interference. An active mouse contact lens electrode (OcuScience) was placed on the cornea, coupled with methylcellulose. Two subcutaneous needles were placed at the lateral canthus and base of the tail and served as reference and ground electrodes, respectively. Impedance was kept below 5 kΩ. Scotopic (dark-adapted) function was recorded in response to 0.3, 1, 3, 10, 30, 100 and 300 mcd*s/m^2^ flashes in a randomly selected eye. Following light adaptation (10 min, 30 cd/m^2^) photopic function was recorded bilaterally in response to 10, 30, 100, 300, 1000, 3000, 10,000 and 25,000 mcd*s/m^2^ flashes.

### Histology, immunofluorescence and EdU analyses

Embryonic heads were fixed with 4% (v/v) paraformaldehyde for 3 h and embedded in paraffin. Immunofluorescence analyses and H&E staining were performed on 10-μm paraffin sections as described (Ashery-Padan et al., 2000). Briefly, sections were immersed in PBS and boiled twice in Unmasking Solution (Vector Laboratories, VE-H-3300). The sections were blocked for 2 h in PBSTG (0.2% Tween 20, 0.2% gelatin in PBS), incubated overnight with primary antibodies, washed with PBSTG, incubated for 2 h at room temperature with the secondary antibodies, washed with PBSTG, and mounted in fluorescent mounting medium (OriGene, E19-18). Antibody sources and dilutions are listed in Table S8.

EdU pulse-chase experiments were carried out by intraperitoneal injection of EdU (50 μg/kg body weight; Lumiprobe, 0540) to pregnant mice 3 h before euthanization. EdU staining was performed by the click-chemistry method (Salic and Mitchison, 2008). The slides were incubated for 35 min before the secondary antibody in solution containing CuSO_4_, L-ascorbic acid (Sigma Aldrich, A4544) and sulfo-cyanine-azide (Lumiprobe, 2330).

For quantification the percentage of EdU from total DAPI-positive cells was determined for the dorsal and ventral regions of interest (Fig. S10). Each data point represents average of two or three sections from same eye. Statistical analyses were performed using GraphPad Prism two-tailed Student's *t*-test as detailed in the figure legends.

Retinal thickness was measured from the H&E staining of each cKO (*n*=3) (Smarcc1 or Smarcc2) and its corresponding control (*n*=3). Ten measurement points were evenly spread at 300-μm steps, five for each side of the retina, beginning from the ONH. The measurement included the entire retina, from the RPE to the retinal nerve fiber layer. Imaging was conducted with an Olympus BX61 fluorescence microscope or an Andor BC43 confocal microscope.

### OCT

OCT was performed to obtain high-resolution, cross-sectional images of the mouse retina. Mice were anesthetized with an intraperitoneal injection of ketamine (85 mg/kg; Vetoquinol) and the relaxing agent xylazine (15 mg/kg; Eurovet Animal Health), and pupils were dilated with Cicloplegicedol (cyclopentolate hydrochloride 1%, Laboratorios Edol, Linda-a-Velha, Portugal) prior to imaging. Retinal scans were acquired using a Heidelberg Spectralis OCT system equipped with a mouse imaging adapter. For each eye, an en-face infrared image of the posterior pole of the eye as well as a series of B-scans covering the central retina were collected in high resolution (HR) mode, providing an axial resolution of up to 3 μm and a lateral (transverse) resolution of approximately 5.7 μm. Volumetric datasets were reconstructed from the acquired scans using the manufacturer's software and further processed for three-dimensional rendering of the retinal layers.

### Geo-seq

The spatial transcriptome of the developing RPE and OS was obtained according to a published Geo-seq protocol (Chen et al., 2017) with minor modifications. Briefly, E12 and E14 mouse embryonic heads were fresh-frozen embedded in OCT (Leica Microsystems, 020108926) and cryosectioned at a thickness of 12 μm. Sections were transferred onto LCM PEN membrane slides (Applied Biosystems™, AB-LCM0522), immediately fixed in ethanol, and stained with 1% (v/v) Cresyl Violet acetate in 75% ethanol solution (Sigma-Aldrich).

From the selected sections, using LCM, we isolated four RPE sections and six OS sections of the E12 embryos (four controls and six *Smarcc1-DCT-cKO*), or three sections of the transverse OS from the E14 embryos (four controls and five *Smarcc1-DCT-cKO*). Samples were collected by laser microdissection and lifted from the PEN membrane slide using the designated cup (Applied Biosystems™, AB-LCM0211). Total RNA pellets were dissolved in a lysis solution (4 M GuSCN), followed by reverse transcription using SuperScript II reverse transcriptase (Invitrogen), and whole transcription amplification with KAPA HiFi HotStart ReadyMix (2×; KAPA Biosystems).

PCR products from the LCM samples were used for RNA-seq library construction. Briefly, PCR products were purified using 0.8:1 ratio AMPure XP beads (Agencourt), quantified with Qubit dsDNA HS Assay Kit (Thermo Fisher Scientific) on an Agilent Tape-station 4200, and a cDNA library was constructed using the Nextera XT Library Prep Kit (Illumina). The E12 samples were sequenced with Novaseq 6000 and the E14 were sequenced on an Illumina Nextseq 2000 instrument both using the 150-bp single-end reads setting.

### Pre-processing of Geo-seq data

The E12 sequencing quality data were evaluated by FastQC and were separately mapped to the mouse GRCm38 genome assemblies using HISAT2 (Pertea et al., 2016) with default settings. Mapping ratio was calculated based on the number of mapped reads and total reads for each sample. All mapped reads were processed by Stringtie (Pertea et al., 2016) to quantify gene expression levels (measured as transcripts per kilobase per million mapped reads) using default parameters.

The E14 RNA-seq data were preprocessed using the Seq2Science pipeline (van der Sande et al., 2023) with the default settings. Briefly, we aligned the reads to the mm10 mouse reference genome using STAR (Dobin et al., 2013). Gene counting was performed using HTSeq (Anders et al., 2015), and transcripts per million normalization was carried out using Salmon (Patro et al., 2017).

### Identification of domain-specific gene expression

To quantify the domain specificity of a gene, an entropy-based strategy was modified from our previous work (Cui et al., 2022). The Jensen–Shannon divergence (JSD) algorithm was used to identify spatial domain-specific genes using the philentropy package (version 0.5.0; https://doi.org/10.21105/joss.00765). For distribution of each gene P1 and predefined pattern P2, the spatial domain specificity score (SSS) between P1 and P2 was defined by converting JSD to a similarity score:

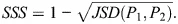


To evaluate the significance of gene specificity in each spatial domain, the SSS was permuted across all samples 1000 times and finally a permutation *P*-value was calculated as the number of times that *SSS*permutation>*SSS*true divided by 1000. Only the significant genes (*P*<0.01) that were specifically enriched in spatial domains were kept for downstream analysis. We used gene set variation analysis (Hanzelmann et al., 2013), an unsupervised method, to estimate variation of domain-specific gene enrichment through the samples of an expression dataset.

### DEGs and clustering analysis

DEG analysis was performed using DESeq2 (Pertea et al., 2016; Love et al., 2014). The count matrix was prefiltered to include only genes with more than ten counts in at least three samples and then subjected to library size normalization and variance stabilizing transformation. The normalized transformed data were then subjected to dimensionality reduction using PCA, and to correlation analysis following hierarchical clustering using the stats and pheatmap package in R to assess sample quality and variance.

To infer all spatial and region-level DEGs, the following statistical tests were performed: (1) each region was compared with all other regions (dorsal versus ventral, dorsal versus axons, ventral versus axons); (2) for each of these regions, a comparison was performed between the control and the *Smarcc1-DCT-cKO*. Genes with adjusted *P*-value lower than 0.05 and log2 (fold change) higher/lower than 0.58/−0.58 were considered DEGs. E12 gene expression clustering was performed on the normalized gene counts with both hierarchical clustering and K-means clustering separately, grouping the samples into the same two groups.

### GSEA of region comparison

Genes were ordered based on their log2(fold change) from the analysis, and were subjected to GSEA software analysis (Subramanian et al., 2005; Mootha et al., 2003). A minimum gene set size of 15 and maximum gene set size of 5000 genes were used, with a *P*-value cutoff of 0.05 and default false discovery rate.

For the GSEA comparison between the E14 OS samples and the E14 RPE control signature genes, we used the K-means clustered DEGs representing the control E14 RPE from our previous paper. Using GSEA, we tested the enrichment of these RPE signature genes across each of the three sampled OS regions: dorsal, ventral and axons (Ovadia et al., 2023).

### AI

Gemini (Google) and Copilot 365 (Microsoft) were used for English language editing and proofreading during the preparation of this manuscript. The final scientific content remains the sole responsibility of the authors.

## Supplementary Material



10.1242/develop.205769_sup1 Supplementary information

Table S1. Metadata and RNA-seq gene counts for E12 LCM samples.

Table S2. Genes with spatially enriched expression in E12 tissue domains.

Table S3. Gene set enrichment analysis (GSEA) of genes enriched in the E12 pRPE-dOS_m_di domain.

Table S4. E14_LCM_Sample data and gene counts: Tab 1 - Details of the 39 E14 LCM samples. Tab 2 - First column shows gene names. The first row is sample names as described in the “Name“ column in tab 1.

Table S5. Differential gene expression analysis of E14 tissue regions: The full outputs of differential expression gene (DEG) calculations performed using DESeq2, corresponding to the experimental scheme shown in Figure 5R.

Table S6. Gene Set Enrichment Analysis (GSEA) for the E14 Smarcc1-DCT-cKO dorsal region.
