# Supplementary material for: Smarcc1 drives optic stalk patterning and optic nerve head astrocyte differentiation
Source: Development. 2026 Aug 13;153(15):dev205769. doi: 10.1242/dev.205769 (PMC13535105; doi:10.1242/dev.205769)
Supplement: Supplementary file 1 [file develop-153-205769-s1.pdf]

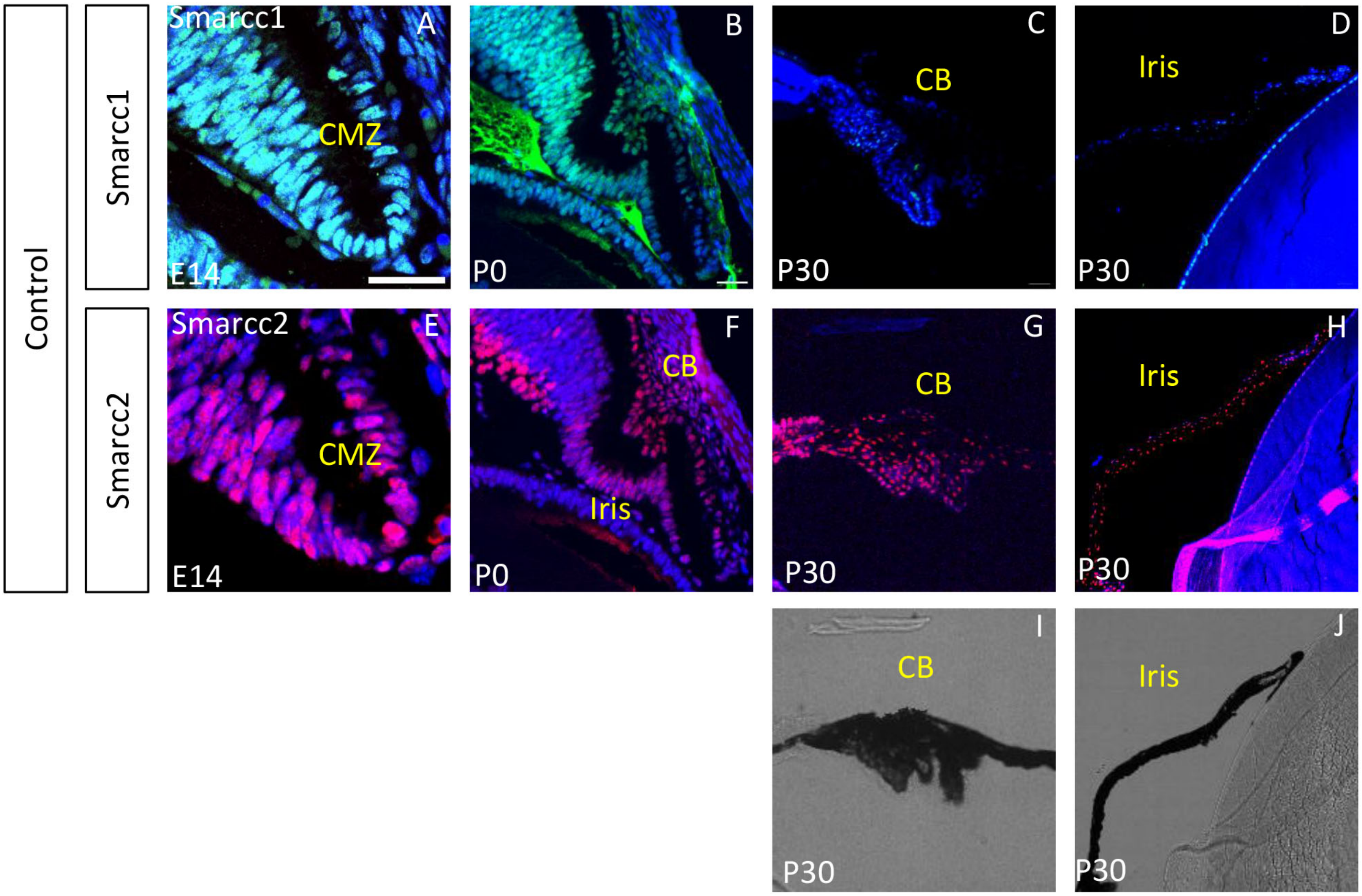

Fig. S1. Stage-specific expression of Smarcc1 and Smarcc2 in the ciliary margin lineages. Immunolabeling for Smarcc1 (A-D), Smarcc2 (E-H) in the developing eye. Expression was examined in the ciliary margin at E14 (A, E), P0 (B, F) and in the iris and ciliary body at P30 (C, D, G, H). Corresponding bright-field images of the P30 iris (I) and P30 ciliary body (J) are included for anatomical reference. Panels B and F represent different fluorescence channels from the same double-labeled tissue section. DAPI was used for nuclear counterstaining in all immunofluorescence panels. Abbreviations: CB, ciliary body; CMZ, ciliary margin zone; CB. Scale bars: 25 µm.

Development • Supplementary information

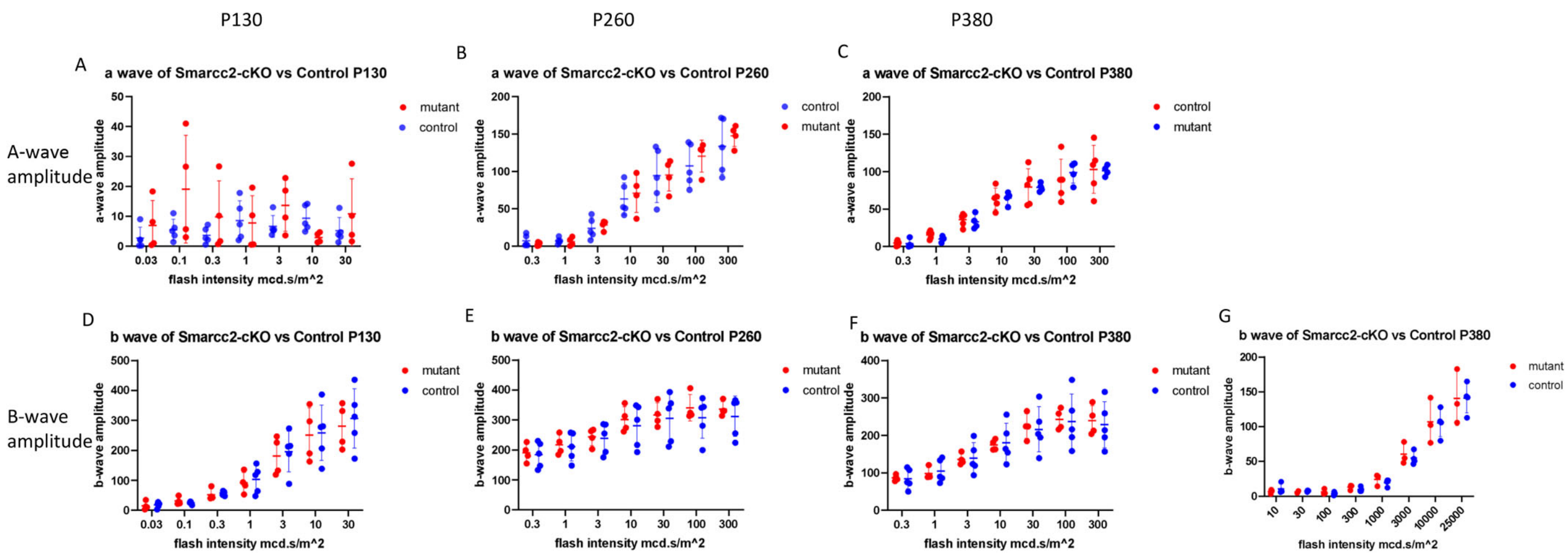

Fig. S2. Electroretinographic responses of Smarcc2-cKO mice show no decline in retinal function with aging.

Scotopic ERG recordings of a-wave amplitudes (A–C) and b-wave amplitudes (D–F) from control and Smarcc2-cKO mice at P130 (A, D), P260 (B, E), and P380 (C, F) (n = 4-5). Photopic b-wave responses from P380 mice are shown in panel G (n = 3-4). Across all ages and conditions, Smarcc2-cKO retinas exhibited normal response amplitudes comparable to controls, indicating preserved retinal function. The graphs in panels C and F are identical to those presented in Fig. 2D,E, included here to facilitate direct comparison across the complete time course.

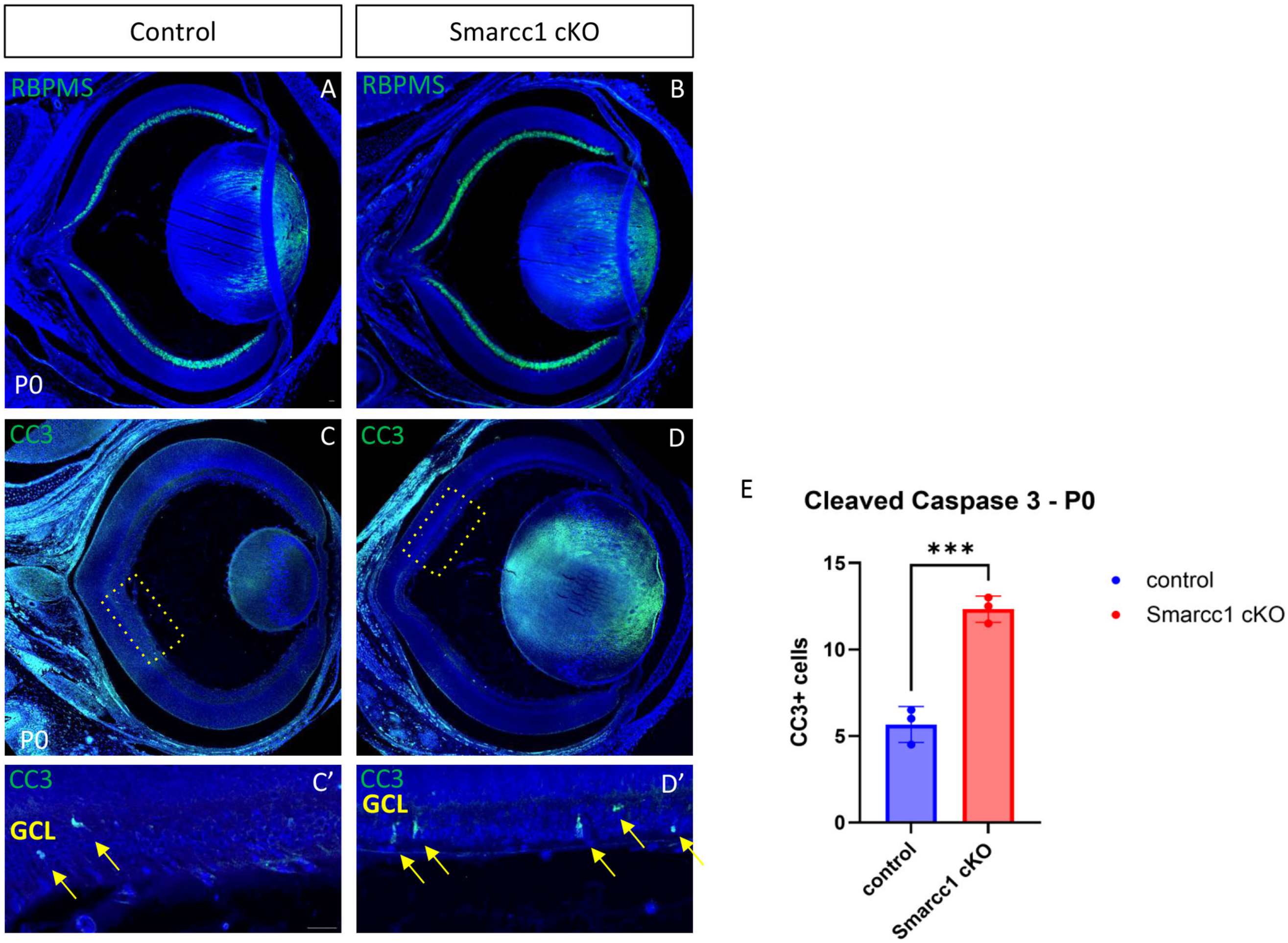

Fig. S3. Smarcc1-cKO increases apoptotic cell death in retinal ganglion cells.

Immunolabeling for the retinal ganglion cell (RGC) marker RBPMS (A, B) and the apoptosis marker cleaved caspase-3 (CC3) (C, D), with corresponding higher-magnification views (C′, D′), in P0 control and Smarcc1-cKO retinas. DAPI was used for nuclear counterstaining. Quantification of $CC3^{+}$ cells in the ganglion cell layer (GCL) is shown in panel E ($n = 3$). Scale bars: 25 µm. Paired two-tailed t-test: $p < 0.001$ (***).

Development • Supplementary information

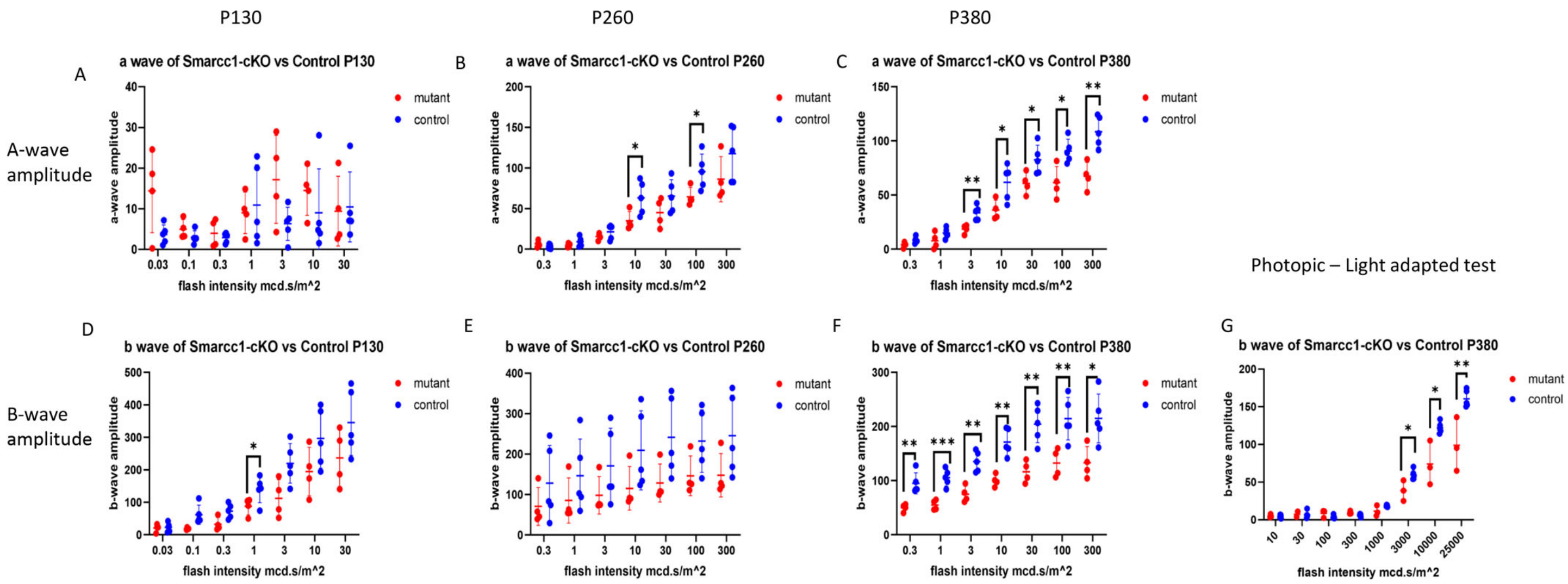

Fig. S4. Smarcc1-cKO retinas exhibit age-dependent decline in ERG responses.

Electroretinographic (ERG) responses from Control and Smarcc1-cKO mutant mice. Scotopic a-wave amplitudes are shown in A–C, and scotopic b-wave amplitudes in D–F, recorded at P130 (A, D), P260 (B, E), and P380 (C, F) (n = 4-5). Photopic b-wave responses at P380 are shown in panel G (n = 3-5). The graphs in panels C and F are identical to those presented in Fig. 3L,M, included here to facilitate direct comparison across the complete time course. Paired two-tailed t-test: $p < 0.01$ (**), $p < 0.001$ (***).

Development • Supplementary information

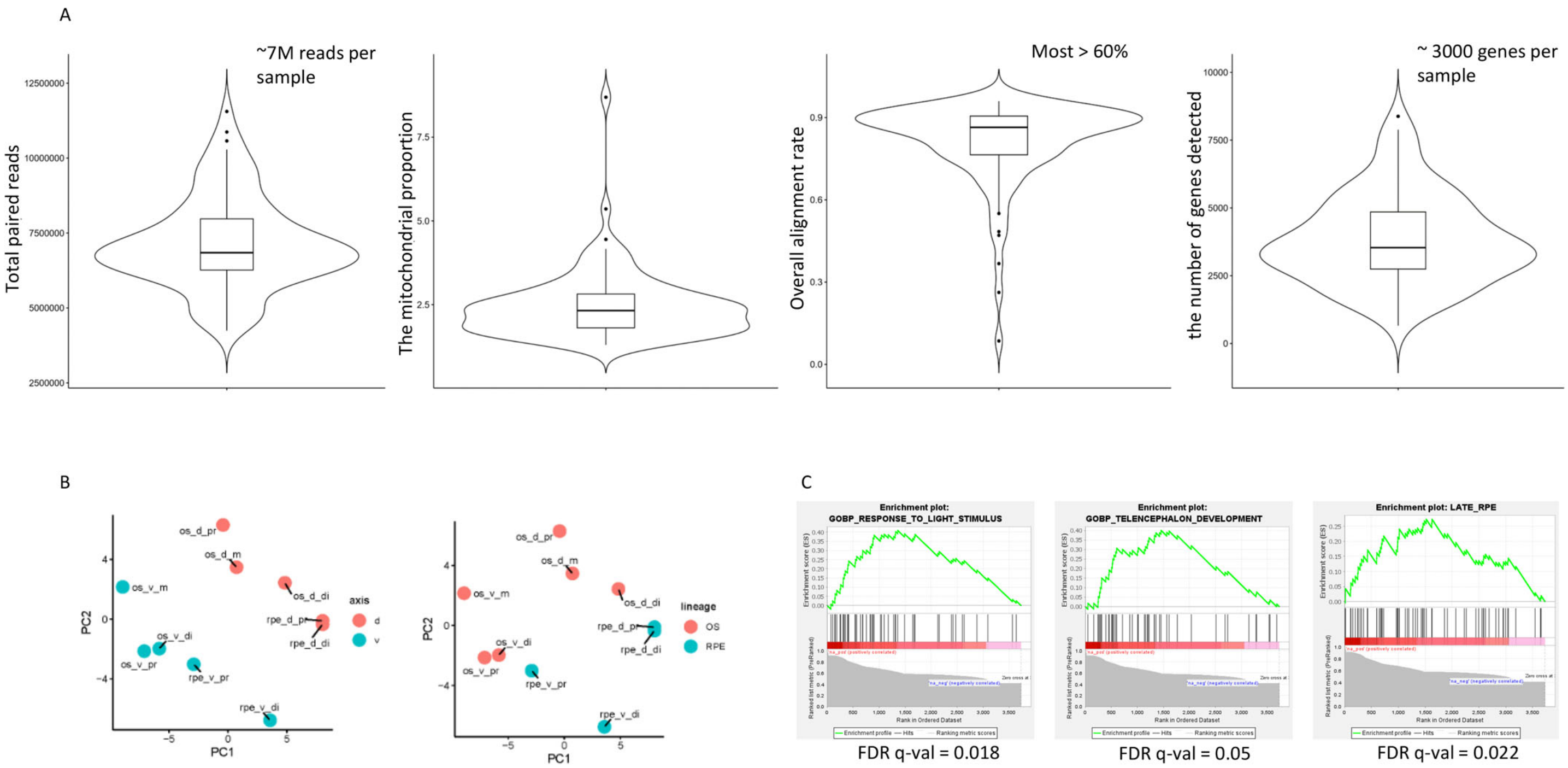

Fig. S5. Quality assessment and transcriptomic characterization of Geo-seq samples.

Violin plots showing key quality control metrics for E12 Geo-seq samples (n = 3–5 per region across 10 regions), including the total number of mapped reads (in millions), mitochondrial gene percentage, overall alignment (mapping) ratio, and the number of detected genes (TPM > 0). (A). Principal component analysis (PCA) of sample transcriptomes (B), organized by dorsal–ventral axis (dorsal = d; ventral = v) or lineage identity (pRPE or OS). Gene Set Enrichment Analysis (GSEA) of the pRPE-dOSm_di 3729-gene set (C), demonstrating enrichment for pathways associated with response to light stimulus, telencephalon development, and late fetal human RPE gene programs (Hu et al., 2019). FDR values for the top enriched pathways are indicated.

Development • Supplementary information

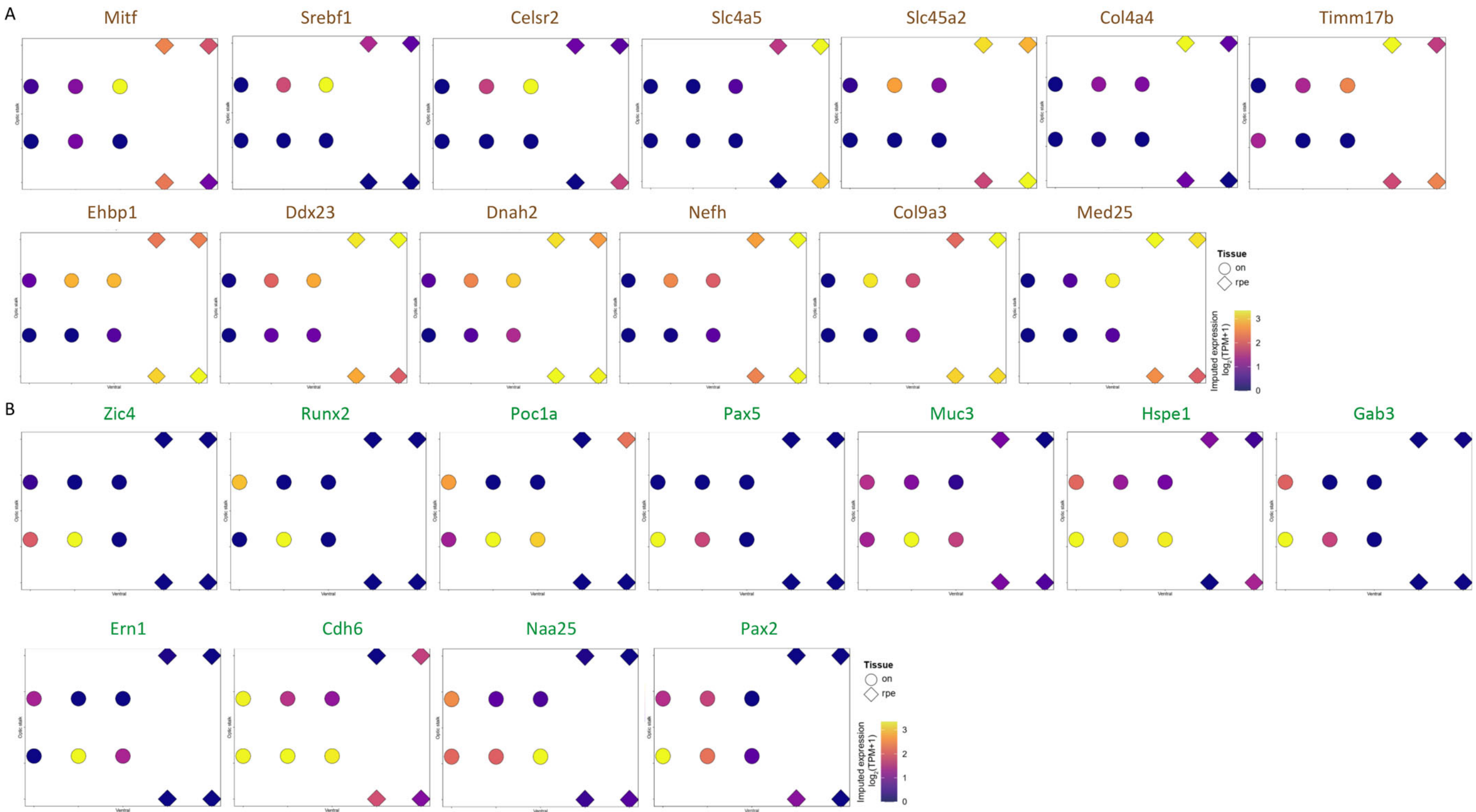

Fig. S6. Spatial expression patterns of domain-enriched genes.

Spatial transcriptomic plots showing imputed expression of representative genes enriched in the pRPE–dOS_m_di domain (A) and the vOS–dOS_pr domain (B) in the developing optic region.

Development • Supplementary information

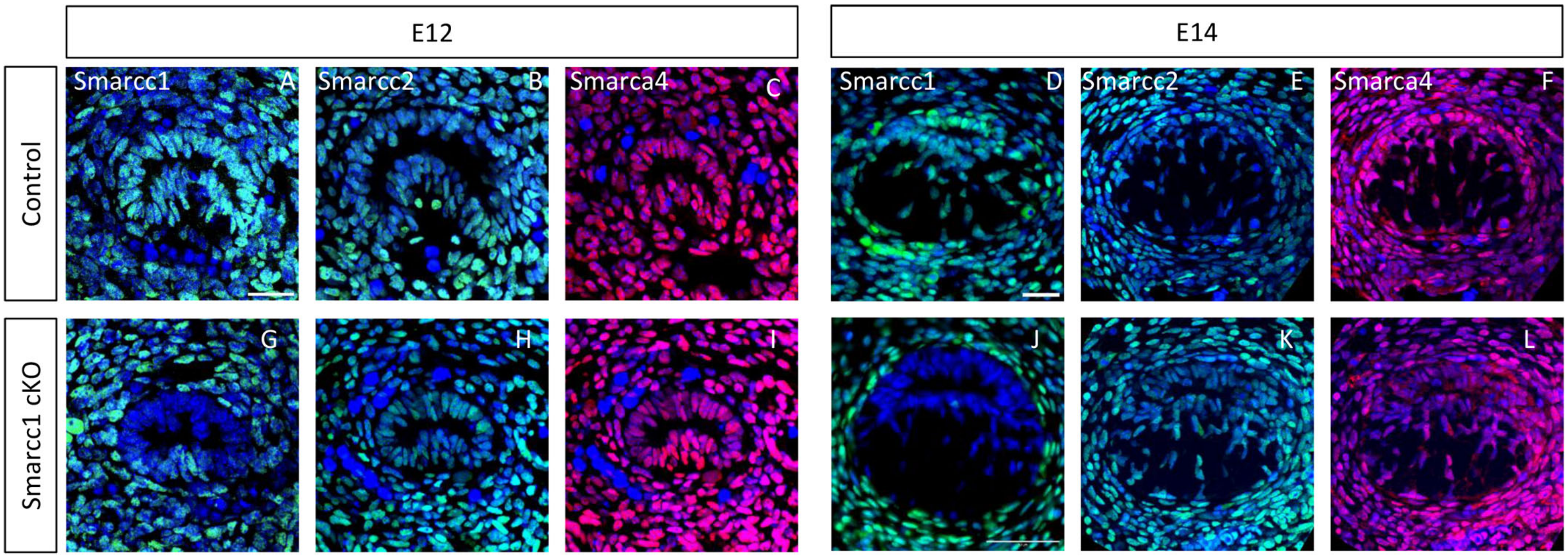

Fig. S7. Smarcc1, Smarcc2, and Smarca4 expression in OS cross-sections at E12 and E14.

Cross-sections of the optic stalk from E12 (A–C, G–I) and E14 (D–F, J–L) mouse embryos labeled with antibodies against Smarcc1 (A, D, G, J), Smarcc2 (B, E, H, K), and Smarca4 (C, F, I, L). Panels E and F, as well as H and I, represent different fluorescence channels from the same double-labeled tissue section. DAPI was used for nuclear counterstaining. Scale bars: 25 µm

Development • Supplementary information

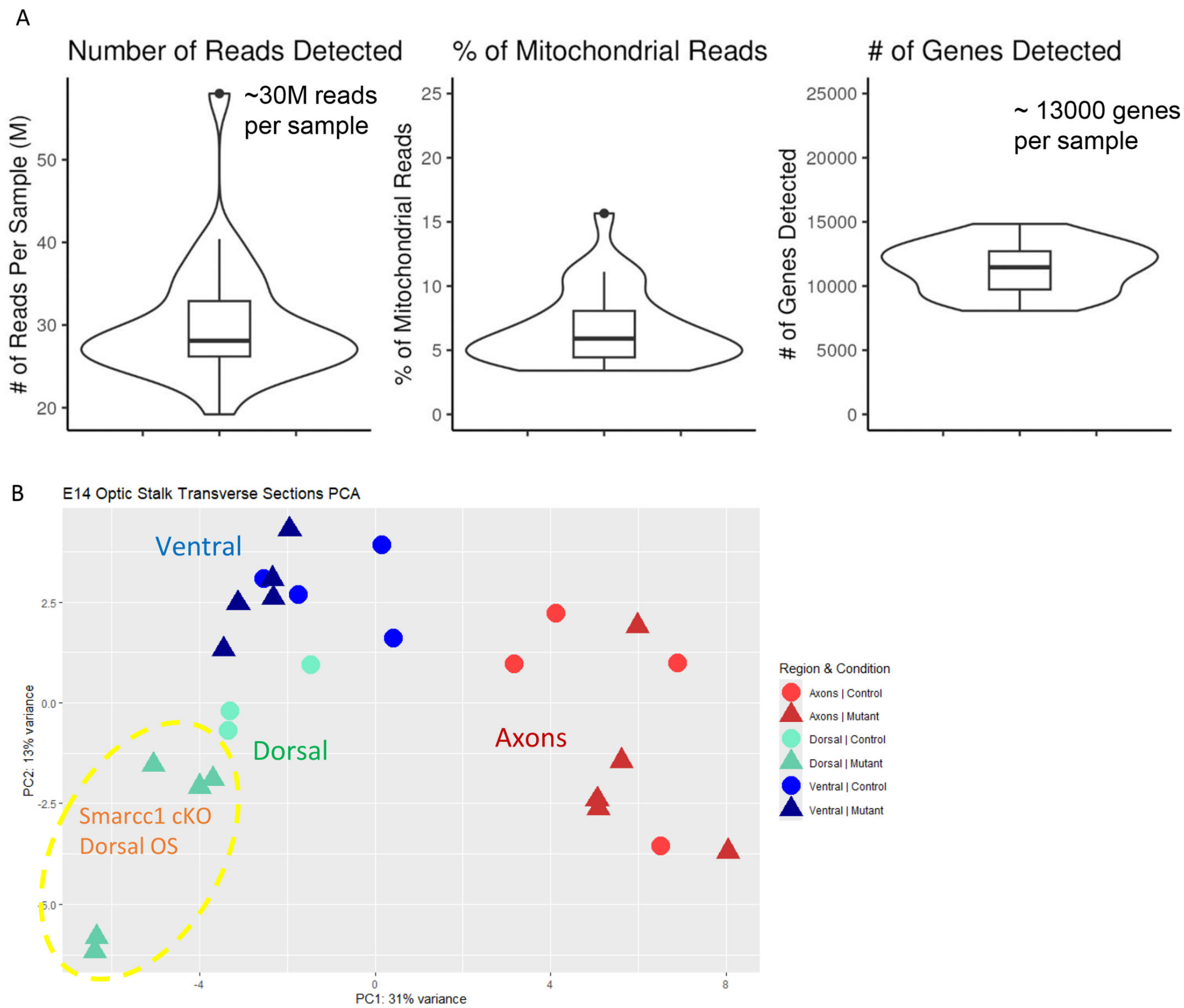

Fig. S8. Quality assessment of Geo-seq samples at E14.

Violin plots (A) summarizing key quality control metrics for E14 Geo-seq samples (n = 3–5 per region across 3 regions), including the percentage of mitochondrial reads, mapping ratio, and the number of detected genes (TPM > 0).

Development • Supplementary information

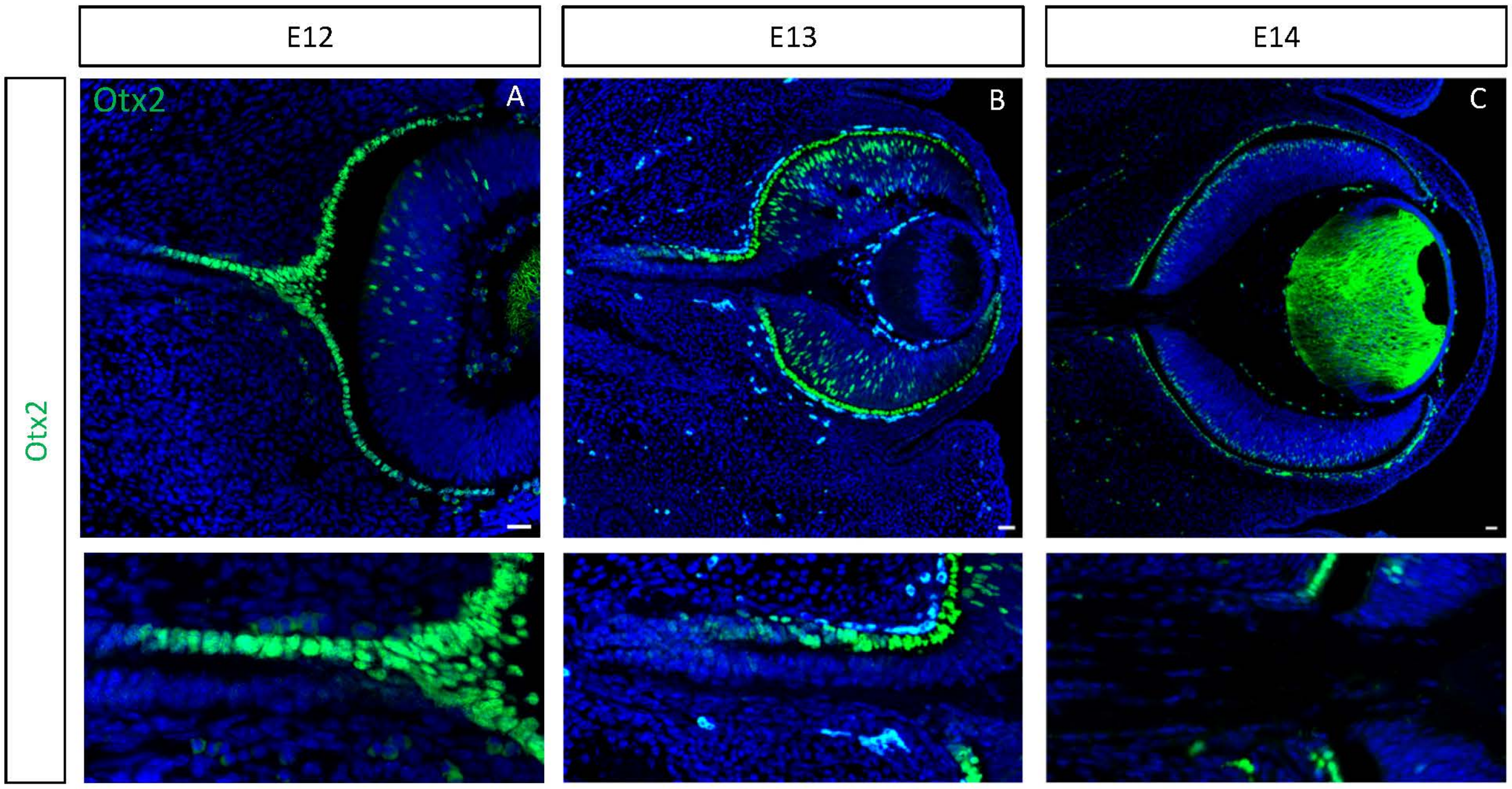

Fig. S9. Otx2 expression during optic stalk differentiation. Antibody labeling for Otx2 in E12 (A), E13 (B) and E14 (C) mouse embryos. The lower panels show higher-magnification views of each time point. DAPI was used for nuclear counterstaining. Scale bars = 25µm.

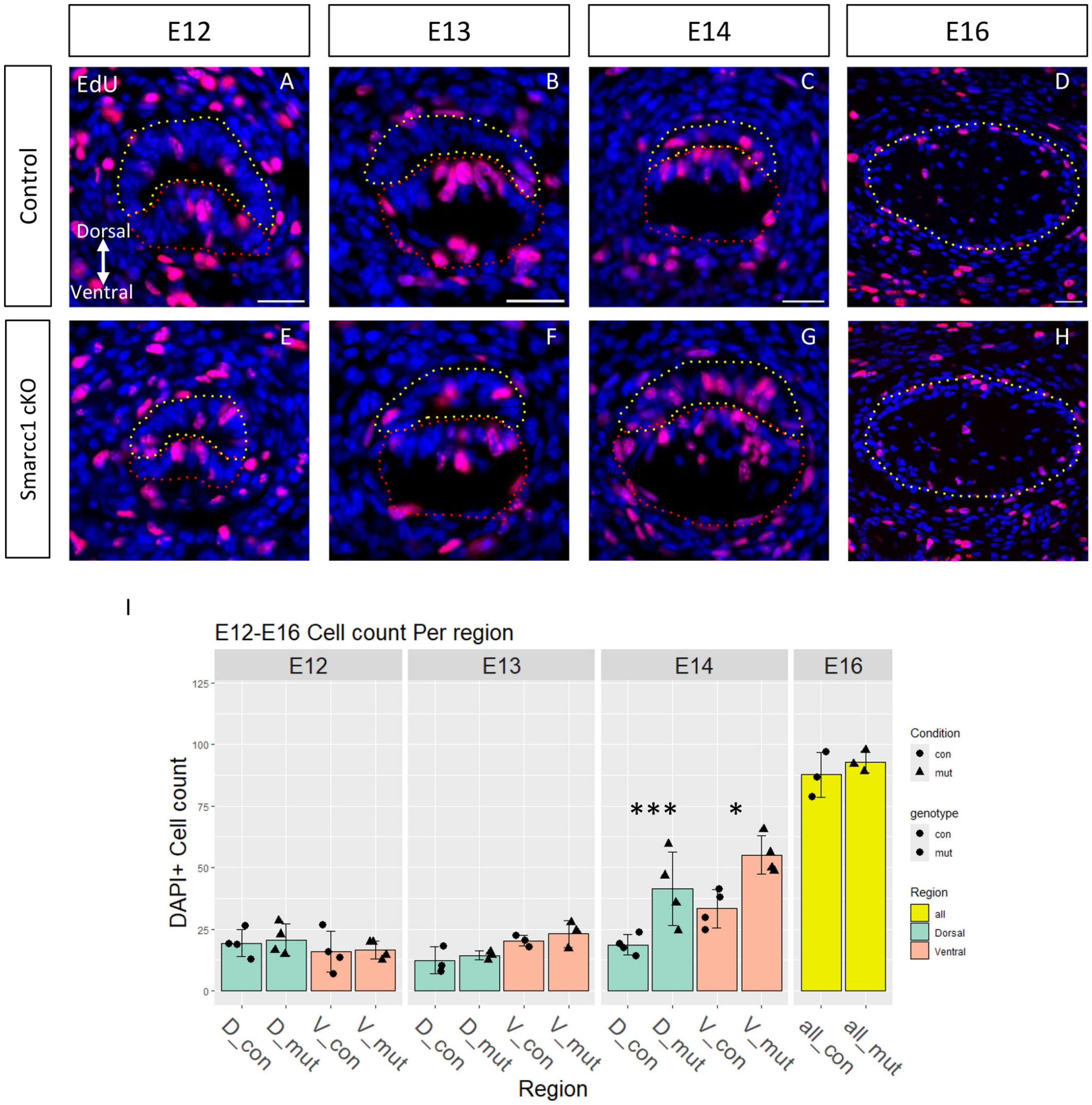

Fig. S10. EdU incorporation in the developing optic stalk of control and Smarcc1-cKO embryos. Representative EdU labeling in control (A–D) and Smarcc1-cKO (E–H) embryos across the dorsal and ventral optic stalk at E12 (A, E), E13 (B, F), E14 (C, G), and E16 (D, H). At E16, quantification was performed across the entire optic stalk, as no dorsal–ventral morphological distinction is present. DAPI was used for nuclear counterstaining and was used to determine total cell counts. Quantification of DAPI^{+} nuclei numbers per region and stage is shown in panel I (n = 4–5). Paired two-tailed t-test: $p < 0.05$ (*), $p < 0.01$ (**), $p < 0.001$ (***). Scale bars: 25µm.

Development • Supplementary information

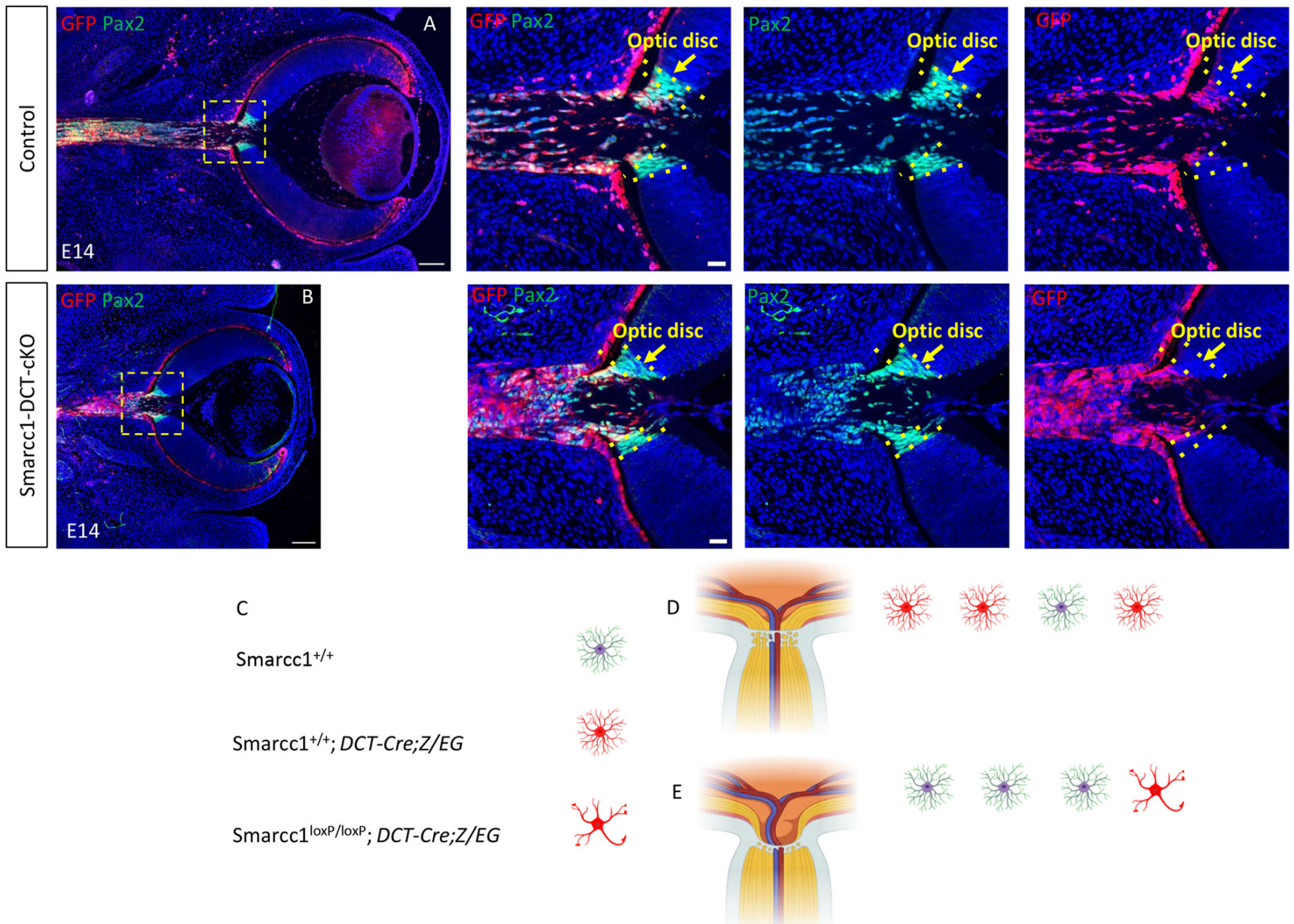

Fig. S11. The *DCT-Cre* transgene is active in a subset of the optic disc progenitor cells. Immunolabeling for eGFP and Pax2 in E14 mouse embryo eye sections of control (A) and Smarcc1-DCT-cKO (B) embryos. Higher-magnification views and single-channel images are shown in the right panels. Co-localization of GFP and Pax2 identifies optic disc progenitor cells in which DCT-Cre is active, whereas Pax2-only cells (between the dashed lines) represent optic disc cells lacking Cre activity. DAPI was used for nuclear counterstaining. (C) Schematic illustration summarizing astrocyte phenotype expected for each of the indicated genotypes. Distribution of retinal astrocyte types in control (D), where the glial lamina forms normally, and in Smarcc1-DCT-cKO (E), where glial lamina formation fails and optic nerve head capping is observed. In control embryos, over 50% of astrocytes show DCT-Cre activity, as indicated by the fluorescent reporter (red), whereas in Smarcc1-DCT-cKO embryos the number of mutant (Cre-positive) astrocytes is reduced. Nonetheless, astrocytes lacking DCT-Cre (green) appear to partially compensate for their loss. Scale bars in A-B = 100µm, Scale bars in magnifications = 25µm.

Development • Supplementary information

Table S1. Metadata and RNA-seq gene counts for E12 LCM samples.

Tab 1 – Sample information: The metadata for each E12 laser-capture microdissection (LCM) sample, including date, genotype, tissue region, sample number, section name, section size, mouse ID, age, experimental condition, and processing batch.

Tab 2 – Gene count matrix: Raw gene expression counts across all samples. The first row lists the sample numbers; the second row lists the sample locations corresponding to each sample number. Rows 3 and below contain individual gene names (Column A) and their respective raw counts.

Available for download at
https://journals.biologists.com/dev/article-lookup/doi/10.1242/dev.205769#supplementary-data

Table S2. Genes with spatially enriched expression in E12 tissue domains.

Tab 1: The 3,729 genes enriched in pRPE–dOS_m_di sections.

Tab 2: The 202 genes enriched in vOS–dOS_pr sections.

For each gene, the following metrics are provided:

Cosine similarity: A measure (0 to 1) of how closely the spatial expression pattern of each gene matches the spatial distribution of the corresponding domain.

Permutation-based p-value: Statistical significance determined by comparing observed similarity to a null distribution generated by random spatial permutations.

Available for download at
https://journals.biologists.com/dev/article-lookup/doi/10.1242/dev.205769#supplementary-data

Table S3. Gene set enrichment analysis (GSEA) of genes enriched in the E12 pRPE–dOS_m_di domain. Genes were ranked according to cosine similarity to the pRPE–dOS_m_di spatial domain. GSEA was performed against selected Gene Ontology and human foetal RPE gene sets.

Tab 1 – GOBP_RESPONSE_TO_LIGHT_STIMULUS gene set

Tab 2 – GOBP_TELENCEPHALON_DEVELOPMENT gene set

Tab 3 – Enrichment against the mid-stage human foetal RPE gene set (Hu et al., 2019).

Tab 4 - Enrichment against the late-stage human foetal RPE gene set (Hu et al., 2019).

Tab 5 – The curated mid (n=568) and late (n=567) human foetal RPE gene sets from Hu et al. 2019 used for GSEA in Tabs 3–4.

Available for download at
https://journals.biologists.com/dev/article-lookup/doi/10.1242/dev.205769#supplementary-data

Table S4. E14_LCM_Sample data and gene counts: Tab 1 – Details of the 39 E14 LCM samples. Tab 2 – First column shows gene names. The first row is sample names as described in the "Name" column in tab 1.

Available for download at
https://journals.biologists.com/dev/article-lookup/doi/10.1242/dev.205769#supplementary-data

Table S5. Differential gene expression analysis of E14 tissue regions: The full outputs of differential expression gene (DEG) calculations performed using DESeq2, corresponding to the experimental scheme shown in Figure 5R.

Tab 1 – Dorsal (Control vs. Smarcc1-DCT-cKO): DEGs between control and Smarcc1 conditional knockout in the dorsal region.

Tab 2 – Ventral (Control vs. Smarcc1-DCT-cKO): DEGs between control and Smarcc1 conditional knockout in the ventral region.

Tab 3 – Axons (Control vs. Smarcc1-DCT-cKO): DEGs between control and Smarcc1 conditional knockout in the axon region.

Tab 4 – Control (Dorsal vs. Ventral): Regional DEG comparison within control samples only.

Tab 5 – Control (Dorsal vs. Axons): Regional DEG comparison within control samples only.

Tab 6 – Control (Ventral vs. Axons): Regional DEG comparison within control samples only.

Available for download at
https://journals.biologists.com/dev/article-lookup/doi/10.1242/dev.205769#supplementary-data

Table S6. Gene Set Enrichment Analysis (GSEA) for the E14 Smarcc1-DCT-cKO dorsal region.

Tab 1 – DESCARTES_ORGANOGENESIS_NEURAL_PROGENITOR_CELLS: GSEA results for the neural progenitor cell gene set.

Tab 2 – DESCARTES_ORGANOGENESIS_MELANOCYTES: GSEA results for the melanocyte gene set.

Tab 3 – RPE SIGNATURE (C1 CLUSTER): GSEA results for the retinal pigment epithelium (RPE) signature gene set from Ovadia et al. (2023).

Available for download at
https://journals.biologists.com/dev/article-lookup/doi/10.1242/dev.205769#supplementary-data

Development • Supplementary information

Table S7. Oligonucleotide primers used for mouse genotyping. List of PCR primers used for mouse genotyping.

Primer	sequance
Cre F primer	CCGCAGAACCTGAAGATGTTC
Cre R primer	TCATCAGCTACACCAGAGACG
Baf155 F primer	TGTCATCCATGAGGAGTGGTC
Baf155 R primer	GGTAGCTCACAAATGCCTGT
Baf170 F primer	CATGGTGGCTCTCCTAAGCAATCCAA
Baf170 R primer	CTGGCTTTGTGTGTGTGTGTTTGTTC
Baf170 Mut Primer	GATGCCTGCTTGCCGAATATCATG
eGFP F primer	AGTTCACCTTGATGCCGTTC
eGFP R primer	CACATGAAGCAGCACGACTT

Development • Supplementary information

Table S8. Antibodies used in this study.

This table lists the specifications, hosts, dilutions, and sources for all primary and secondary antibodies utilized in this study.

	Target	Animal	Company	Serial number	Dilution
Primary Antibodies	Baf155	rabbit	Abcam	ab305037	100
	Baf170	rabbit	Abcam	ab243634	500
	GFP	goat	rockland	600-101-215	500
	Brg1	rabbit	Abcam	ab110641	200
	GFAP (GA5)	mouse	CST	CST - #3670	300
	Myelin Basic Protein (MBP)	rabbit	Abcam	ab40390	1000
	RBPMS	rabbit	Abcam	ab152101	200
	cleaved Caspase3	rabbit	CST	9664	400
	Pax6	Rabbit	Abcam	AB-ab195045	350
	Pax6	mouse	Santa Cruz	sc-53108	50
	Pax2	Rabbit	biolegend	901002	100
	Otx2	Rabbit	Abcam	AB-ab183951	250
	MITF	rabbit	Abcam	ab303530	350
	Sox2	rabbit	CST	CST-23064S	400
	Sox9	rabbit	chemicon	ab5535	200
	NF165 (2H3)	mouse	DSHB	2H3	100
	Nestin	Chicken	Abcam	ab134017	300
Secondary Antibodies	Goat IgG	Donkey	invitrogen	A11058	500
	mouse IgG	Donkey	Abcam	ab175472	500
	Rabbit IgG	Donkey	Abcam	ab150073	500
	Chick IgG	goat	Abcam	ab150169	500

Development • Supplementary information
