# Supplementary material for: Smarcc1 drives optic stalk patterning and optic nerve head astrocyte differentiation
Source: Development. 2026 Aug 13;153(15):dev205769. doi: 10.1242/dev.205769 (PMC13535105; doi:10.1242/dev.205769)
Supplement: Supplementary file 2 [file develop-153-205769_review_history.pdf]

Smarcc1 drives optic stalk patterning and optic nerve head astrocyte differentiation

Nitay Zuk-Bar, Shai Ovadia, Guizhong Cui, Alexey Obolensky, Eyal Banin, Ron Ofri, Naihe Jing and Ruth Ashery-Padan
DOI: 10.1242/dev.205769

Editor: Steve Wilson

Review timeline
Submission to Review Commons: 30 December 2025
Submission to Development: 28 April 2026
Editorial Decision: 16 June 2026

Reviewer 1

Evidence, reproducibility and clarity

Previous work from the Ashery-Padan team has shown that Smarcc1 and Smarcc2, two subunits of SWI/SNF chromatin remodeling complexes, have redundant roles in the specification of the Retinal Pigment Epithelium (RPE) of the optic cup. In the present manuscript, the same team first conform this finding and thereafter show that Smarcc1 has a specific and unique role in the dorsal optic stalk progenitors allowing them to transit from an early RPE identity into Pax2/Sox2-positive astrocyte progenitors. Loss of Smarcc1 results in alteration of the optic nerve head (ONH) morphogensis, glial lamina disorganization, RGC degeneration, and visual decline. The study is based on different experimental approaches including Dct-Cre (RPE) conditional deletion of Smarcc1 and Smarcc2, histology, immunofluorescence, OCT imaging, ERG assays, EdU proliferation analysis, and especially spatial transcriptomics (Geo-seq). The authors conclude that "Smarcc1-mediated chromatin remodelling couples astrocyte progenitor specification and differentiation with local morphogenesis, ensuring long-term retinal function."

Major Comments

This is a well conducted study based on biological replicates and appropriate statistical analysis. The different images are excellent and compelling and the central conclusion that Smarcc1 is essential for dorsal optic stalk-to-astrocyte fate transition is well supported by the combination of developmental marker analysis and in principle by spatial transcriptomics. The latter however requires additional explanations. The reader has to face tables with long list of genes without knowing exactly what the authors have relied upon to identify RPE, OHN or ON.

The authors state that "at E12, prior to optic fissure closure, the dorsal-distal OS shares transcriptional identity with the pRPE, suggesting a common developmental trajectory." This is indeed the case, but it not a particularly new conclusion. It is well known that the dorsal- distal OS express the markers of the RPE and it is its continuation (see PMID: 16965267 for a review). This should be acknowledged and discussed properly.

The authors show that the dorsal OS of Smarcc1-DCTcKO embryos retains RPE identity (in agreement with the initial finding that Smarcc1 and 2 are redundant in this tissue). This finding is however difficult to reconcile with the increased proliferation observed in these cells, given that RPE is known to proliferate at low pace.

Figure 6 R,U shows that astrocyte migration from the ONH into the retina is significantly reduced.

© 2026. Published by The Company of Biologists under the terms of the Creative Commons Attribution License (https://creativecommons.org/licenses/by/4.0/).

However, in Figure 6 S and V GFAP staining of Smarcc1-cKO mice is very similar if not even stronger than in controls. How do the authors explain these findings?

Minor comments

The introduction is a bit long and would benefit from some shortening. It also strange to read some of the conclusions on the study in the first paragraph of the introduction.

Some claims regarding long-term retinal degeneration could be toned down as they have not been investigated in detail.

The discussion is not always fair with previous findings especially on the well documented difference between dorsal and ventral origin of the optic stalk components.

Referees cross-commenting

No additional comments as I believe that there is good agreement on the interest and quality of the study

Significance

This study provides an important mechanistic advance by linking SWI/SNF chromatin remodelling to spatial fate decisions in the developing optic stalk. The discovery of a dorsal- to-ventral temporal wave of astrocyte specification and the unique requirement for Smarcc1 offers conceptual insight into ONH development and may be relevant to congenital optic nerve malformations and glaucomatous vulnerability. The study will be of interest mostly for developmental neurobiologists

Expertise: I have sufficient expertise to evaluate the entire study with particular emphasis on RPE and ONH development.

Reviewer 2

Evidence, reproducibility and clarity

Summary

In this manuscript, Zuk-Bar and colleagues address fundamental questions regarding the cellular and molecular mechanisms controlling optic stalk development and differentiation of optic nerve head astrocytes. The authors examine Smarcc1 and Smarcc2, subunits of the conserved SWI/SNF complex, and identify some partial redundancy, but also distinct requirements for Smarcc1 in optic stalk patterning and optic nerve astrocyte differentiation. These defects lead to altered optic nerve head morphology and impaired retinal ganglion cell survival.
Overall, this was an exciting and very strong study. Data are well presented and images are clear and interpretable. Most of my comments are quite minor.

Specific points:

- Figure 2G, K: The images are meant to demonstrate selective deletion of Smarcc2 from the optic stalk, however, Figure 2K is a bit difficult to evaluate since it is difficult to know if there are any cells in the ONH; the nuclear counterstain appears to be missing as well. Can the authors comment on this?
- Figure 2I, M: The images are interpreted as elevation of Smarcc1 in the ONH, but again, it is difficult to evaluate this, as there appears to be many more cells in the section in 2M compared to 2I. Can the authors comment on this?
- Figure 3E', F': In the text, these panels are referred to as showing pigmented lineages of the RPE iris and ciliary body; only RPE is apparent to me in these zoomed images.
- Figure 4, transcriptomic data: The clustering seems quite clear, and it is agreed that the simplest explanation is that the data suggest a common developmental trajectory for dorsal-

© 2026. Published by The Company of Biologists under the terms of the Creative Commons Attribution License (https://creativecommons.org/licenses/by/4.0/).

distal OS with pRPE. It is also possible, however, depending on the specific genes that are driving the clustering of the dorsal-distal OS with RPE, that there could be extrinsic cues inducing those genes, and that this is the result of proximity to the extrinsic cues, rather than developmental origin or trajectory. This is, of course, a minor point and simply an alternative hypothesis.
- Clarification question regarding domain-specific signature genes and terminology: The domain-specific gene analysis is described as pRPE-enriched and vOS-enriched genes. Based on Figure S5B, dorsal-distal OS has a similar gene signature compared to pRPE. Is dorsal OS being defined as pRPE here? Are the pathways defined as being enriched in pRPE also enriched in and driving clustering with dorsal-distal OS?
- Figure 6G: Is the pigmented patch contiguous with the rest of the RPE? There is not a concern, rather, this is a question out of curiosity.
- Speculative question: The data suggesting the differences between the dorsal and ventral optic stalk at E12/12.5 are quite clear. I am curious if the authors think that it is possible that these dorsal-distal OS cells might move into the eye between E12 and E14, contributing to the RPE as a sheet.

Significance

The study is of conceptual and technical significance and will be of interest to a broad developmental biology, neuroscience, and vision science audience. With respect to conceptual and intellectual significance, it has been known that optic stalk cells differentiate into astrocytes, however the molecular mechanisms governing astrocyte differentiation are not well understood. Further, developmental and morphogenetic events related to the optic stalk (including optic fissure closure), and their relationship to astrocyte differentiation are not well understood. Both of these gaps in knowledge are addressed in this study. From a technical standpoint, this work uses quantitative image analysis and, for the first time, regional transcriptomic analyses to identify optic stalk domains and distinct developmental trajectories. The work in this manuscript uncovers important fundamental aspects of optic stalk and astrocyte development, and it also lays the foundation for future understanding of gene regulatory network control of these processes, which have implications for eye development, retinal health, and vision disorders.

Reviewer 3

Evidence, reproducibility and clarity

Summary:
This study by Zuk-Bar...Ashery-Padan delineates the steps in the development of optic nerve astrocytes, crucial for support of retinal ganglion cell (RGC) axons throughout the lifetime of mice and humans. Here they trace the transition of neuroectodermal derivatives of the embryonic optic cup and optic stalk in the mouse, and identify how Smarcc1 (Baf155) and Smarcc2 (Baf170), scaffolding subunits of SWI/SNF chromatin remodeling complexes, work in tandem to influence stage- and lineage-specific trajectories, using immunohistochemistry and quite beautiful microscopy to trace pigmented lineages of the optic cup. They find that both factors are coexpressed in early retinal pigment epithelium (RPE) and optic stalk progenitors, with Smarcc2 persisting in differentiated RPE and optic nerve astrocytes. They chart changes over several key developmental timepoints embryonically and postnatally, focusing on the optic stalk and optic nerve head, the RPE and retina, and ultimately astrocytes.
Conditional deletion using Dct-Cre revealed reciprocal compensation in pigmented lineages: Smarcc1 loss uniquely caused optic nerve head (ONH) morphogenetic failure, glial lamina collapse, RGC degeneration, and progressive visual decline, nicely measured functionally in aged mice. Spatial transcriptomics and functional assays validated with additional specificity that Smarcc1 drives the transition of dorsal optic stalk progenitors from a pigmented, RPE- like state to astrocyte progenitors by repressing pigment gene programs and enabling Pax2/Sox2 activity.

Major comments:
- Are the key conclusions convincing? Yes
- Should the authors qualify some of their claims as preliminary or speculative, or remove them altogether?
No

© 2026. Published by The Company of Biologists under the terms of the Creative Commons Attribution License (https://creativecommons.org/licenses/by/4.0/).

- Would additional experiments be essential to support the claims of the paper? Request additional experiments only where necessary for the paper as it is, and do not ask authors to open new lines of experimentation.
The conclusions are convincing, and no new experiments are required. The explanation of how the data were gathered for the GeoSeq could be made clearer in the text on P. 10, in Figure 4 E. and F. and Fig. 4 legend: How did they arrive at the conclusion that dorsal OS shares similarities with the ventral OS?

- Are the data and the methods presented in such a way that they can be reproduced?
Yes; however, the Geo-Seq data have been gathered from the very specialized laser ablation of cryosections, with the Geo-seq method, carried out as in Chen et al.. It is a magnificent technique but it is not clear, even with the published methods, whether other labs can perform this approach (though this does not cast aspersions on the method).

- Are the experiments adequately replicated and statistical analysis adequate? Seems rigorous, with some new statistical analyses applied to the Seq data.

Minor comments:
- Specific experimental issues that are easily addressable.
The Geo-Seq is a fantastically precise technique, using Laser Capture Microdissection (LCM). More detail would be welcome: how was the tissue cut out by LCM processed? Please explain more clearly in the Methods, and the areas sampled could be explained better in the text, on the top of P. 13

- Are prior studies referenced appropriately? Yes

- Are the text and figures clear and accurate?
The text is very clear and well written, and the figures are for the most part well constructed and legends clear, except for Fig. 4

- Do you have suggestions that would help the authors improve the presentation of their data and conclusions?

In Fig. 1 F. please put a box around the areas shown enlarged; does ON = optic nerve? How can it be in the same plane as the INL??

Fig. 2 legend: define OS in the title either at end of legend or write OS out in legend title. P. 9, in the text, reference is made to the ciliary body in (Fig. 3E', F') but it is not visible Fig. 3 legend, line 4: "renedring" should be" rendering"
p. 8, third paragraph: please indicate the age examined in the text for Smarcc2-DCT-cKO eyes

Most of the figures are clear except for:
a. Fig. 4G: should the key for Cluster signify ventral and proximal OS (optic stalk),not only Ventral OS, as per the dashed lines in Fig. 4F post-cutting?
b. Figure 7: this summary figure is not clear:
-Add age at the top of A. and B.
-C. and D. boxes, dorsal optic stalk, and astrocyte progenitors, respectively. The boxes labeled Astrocyte specification and Astrocyte differentiation. D. is clear but in C., the genes are all expressed in neuronal progenitors, and most are not mentioned in the text.
-the lines in C. and D. and overall are rather thin and hard to see unless enlarged on a computer screen
-write out GRN in the legend
-the pale blue "V" for the glia at the ONH: should show as disrupted when Smarcc1 is conditionally removed?

Referees cross-commenting

Nice concurrence among the reviewers. I agree with the following two points Reviewer 1. Major Comments 2: "The authors state that "at E12, prior to optic fissure closure, the dorsal-distal OS shares transcriptional identity with the pRPE, suggesting a common developmental trajectory."

© 2026. Published by The Company of Biologists under the terms of the Creative Commons Attribution License (https://creativecommons.org/licenses/by/4.0/).

This is indeed the case, but it not a particularly new conclusion. It is well known that the dorsal-distal OS express the markers of the RPE and it is its continuation (see PMID: 16965267 for a review). This should be acknowledged and discussed properly."
and
Rev 2: Specific Points", fifth point: " Clarification question regarding domain-specific signature genes and terminology: The domain-specific gene analysis is described as pRPE- enriched and vOS-enriched genes. Based on Figure S5B, dorsal-distal OS has a similar gene signature compared to pRPE. Is dorsal OS being defined as pRPE here? Are the pathways defined as being enriched in pRPE also enriched in and driving clustering with dorsal-distal OS?"

With respect to these two Reviewers' comments: authors, in the Results text and Discussion, please address the 'inverse' - that the dorsal-proximal OS is more like the ventral OS (Figure 4 F and G. It is curious that there is such a sharp dividing line in the dorsal OS.

Significance

General assessment: These findings establish a mechanism in which Smarcc1-mediated chromatin remodeling couples astrocyte progenitor specification and differentiation with local morphogenesis. The significance of this work is high; there is emerging emphasis of astroglia at the optic nerve head and their relationship, once disturbed, to glaucoma. Most studies in this area, however, focus on the mature visual system, and here Zuk-Bar et al. elucidate how optic nerve head (ONH) astroglia develop, as the eye develop in relation to optic vesicle lineages. They also show astrocyte progenitor proliferation dynamics in the optic stalk ensuring long-term retinal function.

In the context of the existing literature:

As per glaucoma and other injurious scenarios, they may want to cite and discuss how their results relate to disturbance of astroglia at the optic nerve head after optic nerve injury and indicating an avenue to neuroprotection by acting on these glia in Cameron et al., Nature, 2024, PMID: 38086421).

In addition, the authors aptly point to studies in other cell types and stages matching their observations: that Smarcc2 but not Smarcc1 is expressed in differentiated derivatives of the optic cup, and nicely refer to these studies: The detection of Smarcc2 but not Smarcc1 in differentiated derivatives of the optic cup, in proliferating multipotent progenitors and Smarcc2 in differentiating and mature cell types (Bachmann et al., 2016; Bi-Lin et al., 2021; Zhang et al., 2021).

Interest to other audiences:

These data will be of interest to many labs interested in basic aspects of eye development at the cellular, developmental time line and genomic aspects. And to those studying aspects of disease as mentioned above, especially with regard to the much studied (and maligned) astroglia, here, aspects of astroglia development in the eye/optic nerve are elucidated, to my knowledge, a first using modern approaches.

Author response to reviewers' comments

Manuscript number: RC-2025-03366

Corresponding author: Ruth Ashery-Padan

General Statements
We thank the Review Commons reviewers for their careful evaluation and constructive comments.

© 2026. Published by The Company of Biologists under the terms of the Creative Commons Attribution License (https://creativecommons.org/licenses/by/4.0/).

We greatly appreciate the time and expertise invested in assessing our work. The reviewers' suggestions have substantially improved the clarity, rigor, and presentation of the manuscript. Below, we provide a detailed, point-by-point response to all comments and indicate all modifications made to the manuscript. All changes appear in the revised version.

Point-by-point description of the revisions
General Comment
We thank the reviewer for the positive evaluation and instructive comments. Below we provide detailed responses. All corresponding revisions have been incorporated into the manuscript.

Reviewer 1 – Responses

Major Comments
Response to the Reviewer's Summary Statement
We thank the reviewer for the positive overall summary of our work. We would like to clarify, in response to the summary statement of the reviewer, that in our previous publication we deleted both Smarcc1 and Smarcc2 simultaneously and therefore did not assess their potential redundancy or unique functions. The present study is the first to demonstrate partial functional redundancy as well as a unique, non-redundant requirement for Smarcc1 in dorsal optic stalk astrocyte progenitor specification.

1. GEO-seq explanations and clarity of gene lists
Reviewer comment:
Additional explanations are needed to clarify the GEO-seq analysis; tables show long gene lists without clear guidance.

Response:
We revised Fig. 4 and supplementary figures (S5 and new S6) to improve GEO-seq clarity:

Updated sample labels (d, v, di, pr).
Defined domain groupings explicitly:

Domain 1: pRPE – dOS – m – di
Domain 2: vOS – dOSpr

Corrected tables S1 and S2 accordingly.
Added representative genes for each domain in new Fig. 4I–J.
Added spatial expression examples in new Fig. S6.
Added GSEA results (Fig. 4K, S5, and updated Table S3) of gene set reported, based on single cell analysis by (Hu et al., 2019; https://www.ncbi.nlm.nih.gov/pubmed/31269016) for developing human foetal RPE supporting the enrichment of this gene set the pRPE-dOS_m_di domain.

We hope these extensive revisions make domain identification and GEO-seq interpretation clearer.

2. Prior knowledge of dorsal-distal OS transcriptional similarity to RPE
Reviewer comment:
This is known and should be properly acknowledged.
Response:
We now explicitly cite prior studies (Bharti 2006; Pascalau 2023) describing RPE-like features of the dorsal-distal OS. Our data extend these observations by providing unbiased spatial transcriptomic evidence that dorsal-distal OS clusters with pRPE at a genome-wide level. Manuscript revisions include revised Fig. 4, the following updated wording in the Results and expanded discussion in the

© 2026. Published by The Company of Biologists under the terms of the Creative Commons Attribution License (https://creativecommons.org/licenses/by/4.0/).

Discussion.

Results “Thus, consistent with previous observations that the dorsal–distal optic stalk is continuous with the presumptive RPE (reviewed in (Bharti et al. 2006; Pascalau and Badea 2023)), our spatial transcriptomics at E12 show that dorsal–distal OS samples cluster with pRPE and share a broad pRPE-enriched gene signature, supporting the notion that these cells transiently resemble RPE-progenitor-like transcriptional program before astrocyte specification.”

Discussion “Previous studies have noted that dorsal optic stalk cells exhibit RPE-like characteristics (Silver and Sapiro 1981; Bharti et al. 2006; Pascalau and Badea 2023). Our spatial transcriptomic analysis extends these observations by demonstrating at a genome-wide level that E12 dorsal–distal optic stalk samples cluster with presumptive RPE (pRPE) and display a broad RPE-enriched transcriptional signature. This establishes a dorsal RPE-like identity within the optic stalk and provides systems-level support for continuity between these domains.”

3. Increased proliferation despite preserved RPE identity

Reviewer comment:
How can RPE-like dorsal OS cells proliferate at high levels?
Response:
We agree this may seem counterintuitive. However, SMARCC1/BAF155 normally couples lineage programs to cell-cycle restraint, and its loss is known to affect cell cycle in context dependent manner (for example PMID: 34249931, PMID: 27611684). Thus, Smarcc1 deletion can elevate proliferation without disrupting pRPE identity. Moreover, pRPE cells are naturally proliferative progenitors, and melanin biogenesis initiates during this proliferative phase, making increased cycling compatible with preserved pRPE identity (Ovadia et al., 2023).

4. Apparent discrepancy in Fig. 6 (astrocyte migration vs GFAP intensity)
Reviewer comment:
Reduced migration is observed, yet GFAP signal appears normal or strong.
Response:
The GFAP-positive cells in Smarcc1-cKO retinas are non-recombined astrocytes that escaped Dct-Cre activation (see revised Fig. S11). This is concluded based on lineage tracing using the Z/EG reporter. In the Smarcc1-DCT-cKO the lineage traced astrocytes GFP+) are substantially reduced from total GFAP labeled astrocytes. From this we conclude that the normal cells, in respect of Smarcc1, compensated for the reduced number of Smarcc1 deficient astrocytes.

To clarify this, we revised the respective sections in the result (last paragraph of results) and added a schematic to the lineage tracing approach Fig. S11C.

Minor Comments

5. Introduction too long; contains conclusions
Response: We shortened and reorganized the introduction, removing conclusions from the opening paragraph and adding concluding paragraph to the introduction.

6. Claims about long-term degeneration should be moderated
Response: The text now states that long-term decline is likely secondary to early ONH abnormalities, not necessarily due to persistent Smarcc1 loss.
Updates appear in the Results and Discussion.

Results: “The age-related reduction in retinal thickness and the progressive decline in retinal function are likely driven primarily, but not exclusively, from ONH abnormalities and surrounding retinal atrophy caused by Smarcc1 deficiency.”

© 2026. Published by The Company of Biologists under the terms of the Creative Commons Attribution License (https://creativecommons.org/licenses/by/4.0/).

Discussion page 18 **(in bold is what we have added):** "The alterations in the optic nerve head seem to result in non-cell-autonomous effect on the retina, as it was accompanied by early loss of ganglion cells and reduced retinal activity based on ERG analysis in aged mice. **Nonetheless, future studies using a Cre driver restricted to the RPE, but not the optic stalk, will be necessary to determine whether Smarcc1 expression in the embryonic RPE contributes to RPE differentiation and maintained functions in the adult."**

7. Discussion should better reflect earlier dorsal–ventral OS literature
Response: We expanded relevant discussion sections to ensure a balanced integration of prior work (see response to point 2 above).

<u>**Reviewer 2 – Responses**</u>

General Summary
We thank Reviewer 2 for the thorough and positive assessment of our work and the constructive comments. Below we address all comments.

1. Figure 2G/K — unclear ONH due to lack of nuclear counterstain
Response: We repeated immunostaining with optimized DAPI, added high-magnification insets (Fig. 2G, 2K, panels on the right).

2. Figure 2I/M — difficulty comparing Smarcc1 levels
Response: We repeated staining with stronger DAPI and added magnified insets (Fig. 2I-M, panels on the right). The updated images remove earlier confounding differences in section quality.

3. Figure 3-absence of ciliary body/iris in zooms
Response: We added enlarged crops clearly showing the iris and ciliary body (revised Fig. 3E''', E'''', F''',F'''').

4. Alternative hypothesis for pRPE–dOS clustering
Response: We incorporated this into the Discussion, noting that shared transcriptional identity may reflect shared lineage origins, change in tissue morphology, or shared exposure to local inductive cues. We addressed these options in the discussion:

"Previous studies have noted that dorsal optic stalk cells exhibit RPE-like characteristics (Silver and Sapiro 1981; Bharti et al. 2006; Pascalau and Badea 2023). Our spatial transcriptomic analysis extends these observations by demonstrating at a genome-wide level that E12 dorsal–distal optic stalk samples cluster with presumptive RPE (pRPE) and display a broad RPE-enriched transcriptional signature. This establishes a dorsal RPE-like identity within the optic stalk and provides systems-level support for continuity between these domains. The transcriptional similarity may reflect a shared lineage trajectory, possibly due to transient epithelial cell rearrangements and cell stretching occurring during optic cup morphogenesis, processes documented in teleost embryos but not yet demonstrated in mammals (Kwan et al. 2012; Heermann et al. 2015; Eckert et al. 2019; Moreno-Marmol et al. 2021). Alternatively, the similarity in gene expression may arise from shared exposure to local inductive cues (reviewed in (Morcillo et al. 2006)) that activate similar gene regulatory networks independent of lineage origin. Definitive resolution of these relationships will require lineage-tracing and live-imaging approaches to distinguish between lineage continuity and inductive convergence during optic stalk formation."

5. Domain-specific gene sets: clarifying terminology
Response: To clarify this we now defined domain groupings explicitly:

Domain 1: pRPE – dOS – m – di

© 2026. Published by The Company of Biologists under the terms of the Creative Commons Attribution License (https://creativecommons.org/licenses/by/4.0/).

Domain 2: vOS – dOSpr

6. Pigmented patch continuity (Fig. 6G)
Response: We observed that the pigmented region is contiguous with the RPE near the distal optic nerve.

7. Possible contribution of dorsal-distal OS cells to RPE
Response: We now discuss this explicitly in the Discussion, referencing teleost data suggesting epithelial rearrangement and emphasizing the need for lineage tracing in mammals (see response to comment 4 of the reviewer).

Minor corrections
The following were corrected:

Typo "Smracc" → "Smarcc".
Added DAPI references in all figures where DAPI is used for nuclei counter staining.
Corrected Fig. S2G artifact.
Corrected PCA reference (S8B).
Corrected the reference to the labeling in Fig. 5.
Corrected missing word ("based on").
Updated terminology and formatting throughout.

Reviewer 3 – Responses

General Summary
We thank Reviewer 3 for the positive and constructive evaluation of the manuscript. Below we address the reviewer's helpful suggestions.

Major Comments
1. Clarity on GEO-seq sampling and interpretation
Response: We revised Fig. 4 and supplementary figures (S5 and new S6) to clarify LCM sampling, spatial orientation, and clustering results and our interpretations: These revisions include:

Updated sample labels (d, v, di, pr).
Defined domain groupings explicitly:

Domain 1: pRPE – dOS – m – di
Domain 2: vOS – dOSpr

Corrected tables S1 and S2 accordingly.
Added representative genes for each domain in new Fig. 4I–J.
Added spatial expression examples in new Fig. S6.
Added GSEA results (Fig. 4K, S5, and updated Table S3) of gene set reported, based on single cell analysis by (Hu et al., 2019; https://www.ncbi.nlm.nih.gov/pubmed/31269016) for developing human foetal RPE supporting the enrichment of this gene set the pRPE-dOS_m_di domain.
We hope these extensive revisions make domain identification and GEO-seq interpretation clearer.

2. GEO-seq reproducibility
Response: We added specific methodological details (LCM slides, lysis buffer, section thickness, RNA handling) to the Methods, supplementing the extensive Nature protocol (Chen et al., 2017).

3. Clarity of sampled regions (page 13)
Response: We describe the number, orientation, and boundaries of OS and pRPE sections collected

© 2026. Published by The Company of Biologists under the terms of the Creative Commons Attribution License (https://creativecommons.org/licenses/by/4.0/).

at E12 and E14. We updated sample labels (d, v, di, pr) and corrected the respective supplementary tables S1-S2 accordingly

4. Figure 4 overall clarity

Response: We substantially revised Fig. 4, improving visual clarity, color coding, gene examples, and domain layout (see details in response to comment 1).

Minor Comments

5. Fig. 1F — box around enlarged region
Added.

6. Define OS in Fig. 2 legend
Corrected.

7. Ciliary body visibility in Fig. 3
Fixed by adding enlarged panels (E'''-E'''';F'''-F'''').

8. Fig. 3 legend typo "renedring"
Corrected.

9. Page 8 — add age of Smarcc2-cKO eyes
Added the specific ages.

10. Fig. 4G — clarify cluster label
We defined domain groupings explicitly:

Domain 1: pRPE – dOS – m – di
Domain 2: vOS – dOSpr

11. Fig. 7 improvements
Response: We revised the figure 7 extensively as follows:
Added developmental stage labels
Replaced some of the illustration with immunolabeling and histology images to better represent key findings. The figure legends were corrected accordingly.
We added reference to this figure and to the gene regulatory network in the discussion:

"Our data further indicate that the posterior optic nerve head region is delayed in acquiring astrocytic identity and instead retains RPE-like transcriptional features after the adjacent optic stalk regions have committed to the astrocyte lineage. This conclusion is supported by the expression patterns of Pax6, Mitf, Otx2 (Figs 4, S8, Fig. 7A-B), suggests that the posterior ONH may represent a transitional or regulatory zone, where cell fate decisions are temporally postponed, possibly to coordinate with late-stage events such as optic cup morphogenesis, vascular invasion, and glial lamina formation as well as postnatal growth at the posterior optic nerve head (Bernstein et al. 2020)."

"First, it is required for the specification of dorsal optic stalk cells into astrocytes. In its absence, these cells fail to initiate astrocyte differentiation and instead retain progenitor-like, RPE-like characteristics, marked by elevated proliferation and expression of Mitf, Otx2, and Pax6 and failure to upregulate Pax2 (Fig. 7B). Although dorsal cells initially expand due to increased proliferation, this is not sustained, and the pigmented cell patch observed at P0 is small and does

© 2026. Published by The Company of Biologists under the terms of the Creative Commons Attribution License (https://creativecommons.org/licenses/by/4.0/).

not integrate into the ONH structure. We further detected ectopic NF165 expression in the dorsal optic stalk. Consistent with this, RNA-seq analysis revealed reduced Hes1 expression and elevated expression of several proneuronal genes in the Smarcc1 mutant optic stalk. Hes1 a Notch/Hedgehog-responsive transcriptional repressor documented to inhibit the proneural genes and to contribute to optic stalk and optic cup boundary maintenance (Kageyama et al. 2008; Wall et al. 2009; Bosze et al. 2020). This finding therefore supports a model in which Smarcc1 plays a role in repression of neuroepithelial identity, at least partly through Hes1, and that this contributes to the timely acquisition of glial fate (Fig. 5AA, Fig. 7B). "

12. Discussion — relevance to optic nerve injury
Response: We added a paragraph integrating recent findings on ONH astrocyte dysfunction in optic nerve injury and degeneration.

"Astrocyte dysfunction in the adult optic nerve also contributes to glaucoma models and post-injury scenarios, where ONH astroglial disturbances exacerbate RGC loss (Lee et al. 2025). Emerging work identifying distinct ONH astrocyte subtypes and exploring their targeted manipulation in glaucoma further highlights the need to understand the developmental origins and molecular regulation of these glial populations (Cameron et al. 2024). Thus, elucidating the developmental mechanisms governing ONH astrocyte formation is critical for understanding optic nerve dysfunction and for informing future neuroprotective strategies."

Cross-Commenting -
We integrated the highlighted points:

1. Clarification of pathway enrichment driving pRPE–dOS clustering (see response to reviewer 1 point 1).

2. Acknowledgement of prior dorsal-distal OS literature (see response to reviewer 1 point 2).

Original submission

First decision letter

MS ID#: dev.205769

MS TITLE: Smarcc1 is essential for the patterning of the optic stalk and differentiation of the optic nerve head astrocytes

AUTHORS: Nitay Zuk-Bar; Shai Ovadia; Guizhong Cui; Alexey Obolensky; Eyal Banin; Ron Ofri; Naihe Jing; Ruth Ashery-Padan

Dear Ruth,

Thank you for sending your manuscript to Development through Review Commons.

I am happy to tell you that the referees are happy with your revisions and your manuscript has been accepted for publication in Development, pending our standard publication integrity checks.

Reviewer 1

SUMMARY OF THE ADVANCE MADE IN THIS PAPER AND ITS POTENTIAL SIGNIFICANCE TO THE FIELD

This manuscript from Ashery-Padan lab investigates the role of the chromatin-remodeling factor Smarcc1 during optic nerve development. The study takes advantage of conditional mouse mutants, lineage tracing, spatial transcriptomics, and functional analyses, to show that the dorsal

© 2026. Published by The Company of Biologists under the terms of the Creative Commons Attribution License (https://creativecommons.org/licenses/by/4.0/).

optic stalk initially exhibits a transient RPE-like transcriptional identity, whereas the ventral optic stalk acquires astrocyte progenitor characteristics earlier. Smarcc1 is required for the transition of dorsal optic stalk cells from this pigmented progenitor state into Pax2/Sox2-positive astrocyte progenitors. Smarcc1-deficient mice develop severe optic nerve head malformations, defective glial lamina formation, impaired astrocyte migration, and progressive retinal ganglion cell degeneration associated with visual dysfunction. The study shows a previously unrecognized spatiotemporal organization of astrocyte specification within the optic stalk and identifies Smarcc1 as a key regulator coupling cell fate specification, morphogenesis, and long-term optic nerve integrity.

SUGGESTIONS TO AUTHORS

The authors have addressed most of the concerns raised during previous revision. The represent a solid developmental neurobiology manuscript with well supported novel message. The lineage relationships underlying the dorsal optic stalk/RPE-like state is still somewhat unresolved but this does not humper the publication of the manuscript in the present form.

Reviewer 2

SUMMARY OF THE ADVANCE MADE IN THIS PAPER AND ITS POTENTIAL SIGNIFICANCE TO THE FIELD

In this manuscript, Zuk-Bar and colleagues address fundamental questions regarding the cellular and molecular mechanisms controlling optic stalk development and differentiation of optic nerve head astrocytes. The authors examine Smarcc1 and Smarcc2, subunits of the conserved SWI/SNF complex, and identify partial redundancy and distinct requirements for Smarcc1 in optic stalk patterning and optic nerve astrocyte differentiation. The defects caused by loss of Smarcc1 lead to altered optic nerve head morphology and impaired retinal ganglion cell survival.

SUGGESTIONS TO AUTHORS

In this revised manuscript, the authors have adequately responded to all of my previous questions and comments. I have no further comments.

Reviewer 3

SUMMARY OF THE ADVANCE MADE IN THIS PAPER AND ITS POTENTIAL SIGNIFICANCE TO THE FIELD

The authors should be congratulated on this rich and well documented study, delineating how Smarcc1 and Smarcc2 subunits of SWI/SNF chromatin remodeling complexes act to influence the transition of dorsal optic stalk progenitors from RPE-like to astrocytic in the optic nerve head, followed by glial lamina formation. This is a welcome chronicle of normal development of the eye and the optic nerve head with its specialized glial array, a vulnerable site in many eye disorders.

© 2026. Published by The Company of Biologists under the terms of the Creative Commons Attribution License (https://creativecommons.org/licenses/by/4.0/).
